# Modern artificial intelligence and large language models in graduate medical education: a scoping review of attitudes, applications & practice

**DOI:** 10.1186/s12909-025-07321-5

**Published:** 2025-05-20

**Authors:** Basil George Verghese, Charoo Iyer, Tanvi Borse, Shiamak Cooper, Jacob White, Ryan Sheehy

**Affiliations:** 1 Education for Health Professions Program, School of Education, Johns Hopkins University, 2800 N Charles St, Baltimore, MD 21218 USA; 2 Internal Medicine Residency Program, Rochester, NY USA; 3 West Virginia University, Morgantown, WV USA; 4 Internal Medicine, Parkview Health, Fort Wayne, IN USA; 5 Internal Medicine, Rochester General Hospital, Rochester, NY USA; 6 Welch Medical Library, Johns Hopkins University, Baltimore, MD USA; 7 School of Medicine, University of Kansas Medical Center, Salina, KS campus, Kansas City, KS USA

**Keywords:** Artificial intelligence in graduate medical education, AI integration in medical residency training, Applications of AI in GME, Large language models in medical education, Perceptions of AI in medical training, AI-driven assessments in medical education

## Abstract

**Background:**

Artificial intelligence (AI) holds transformative potential for graduate medical education (GME), yet, a comprehensive exploration of AI’s applications, perceptions, and limitations in GME is lacking.

**Objective:**

To map the current literature on AI in GME, identifying prevailing perceptions, applications, and research gaps to inform future research, policy discussions, and educational practices through a scoping review.

**Methods:**

Following the Joanna Briggs Institute guidelines and the PRISMA-ScR checklist a comprehensive search of multiple databases up to February 2024 was performed to include studies addressing AI interventions in GME.

**Results:**

Out of 1734 citations, 102 studies met the inclusion criteria, conducted across 16 countries, predominantly from North America (72), Asia (14), and Europe (6). Radiology had the highest number of publications (21), followed by general surgery (11) and emergency medicine (8). The majority of studies were published in 2023. Several key thematic areas emerged from the literature. Initially, perceptions of AI in graduate medical education (GME) were mixed, but have increasingly shifted toward a more favorable outlook, particularly as the benefits of AI integration in education become more apparent. In assessments, AI demonstrated the ability to differentiate between skill levels and offer meaningful feedback. It has also been effective in evaluating narrative comments to assess resident performance. In the domain of recruitment, AI tools have been applied to analyze letters of recommendation, applications, and personal statements, helping identify potential biases and improve equity in candidate selection. Furthermore, large language models consistently outperformed average candidates on board certification and in-training examinations, indicating their potential utility in standardized assessments. Finally, AI tools showed promise in enhancing clinical decision-making by supporting trainees with improved diagnostic accuracy and efficiency.

**Conclusions:**

This scoping review provides a comprehensive overview of applications and limitations of AI in GME but is limited with potential biases, study heterogeneity, and evolving nature of AI.

## Introduction

Modern AI refers to data-driven systems that perform tasks requiring human intelligence, such as learning, natural language processing (NLP), pattern recognition, problem-solving, and autonomous decision-making [1]. These systems, which utilize techniques like machine learning, deep learning, and neural networks, continuously adapt and improve with large datasets. This contrasts with traditional AI systems, which rely on static, rule-based logic and require manual configuration and oversight, lacking the adaptive, data-driven learning capabilities of modern models [2]. For instance, earlier systems required faculty to manually interpret and summarize resident performance data, whereas modern AI tools like large language models can automate this analysis, offering timely insights and reducing administrative burden [3]. Modern AI is transforming educational methodologies from a one-size-fits-all approach to personalized learning tailored to individual strengths and needs [4] and is increasingly being considered to address these diverse training requirements [5].

Graduate Medical Education (GME) plays a crucial role in developing healthcare provider’s skills and competencies to meet the evolving healthcare landscape [6] While AI holds transformative potential, its integration into graduate medical education (GME) must be considered within the context of the evolving challenges that GME faces [7]. These challenges—such as adapting to changing healthcare paradigms and maintaining relevant curricula—necessitate innovative solutions, including AI, to enhance educational outcomes and better prepare future medical professionals [8].

However, despite its growing presence in healthcare, the integration of AI into GME remains underexplored. While individual studies highlight AI’s applications in specific areas, such as clinical decision support [9] or simulation-based training [10], a comprehensive understanding of its impact across GME programs is lacking.

Previous scoping reviews have examined the role of AI in undergraduate medical education [11], surgery [12], and pharmacy [13], showcasing its promise in these domains. However, GME—a critical phase of physician training—has yet to be systematically reviewed. This gap in the literature underscores the need for a scoping review to map the existing evidence on AI’s applications, identify perceptions and barriers, and pinpoint research gaps in this field.

Following the Joanna Briggs Institute (JBI) guidelines [14], this scoping review seeks to provide a foundation for future research, policy discussions, and educational practices regarding AI’s role in GME. By doing so, it aims to ensure that AI is effectively leveraged to enhance the training and development of future physicians.

## Methodology

Our scoping review followed the Joanna Briggs Institute (JBI) guidelines [15] and used the Preferred Reporting Items for Systematic Reviews and Meta-Analysis extension for Scoping Reviews (PRISMA-ScR) [16] checklist. We registered our protocol on the Open Science Forum [17].

### Eligibility criteria

#### Population

Original peer-reviewed studies explicitly mentioning Graduate Medical Education (GME) were included. Eligible studies focused on residents (across any specialty), postgraduate trainees, fellows, or faculty.

#### Intervention

AI interventions including a range of technologies such as large language models (LLMs), natural language processing (NLP), recurrent neural networks (RNNs), artificial neural networks (ANNs), convolutional neural networks (CNNs), and other related approaches.

#### Comparator

Given the nature of the scoping review, specific comparators were not required.

#### Outcomes

The primary outcomes of interest were AI’s perceptions, application, and limitations in GME.

### Study selection and extraction

We performed a comprehensive search across multiple databases, including MEDLINE-Ovid, ERIC, EMBASE, CINAHL, Web of Science Core Collection, Compendex, Scopus, and IEEE Xplore, from inception to February 2024. These databases were selected to ensure broad coverage of medical education literature, including specialized fields such as healthcare technology (IEEE Xplore) and educational research (ERIC), while also capturing interdisciplinary perspectives from both medical and engineering disciplines. This broad selection was intended to maximize the inclusivity of relevant studies across diverse healthcare sectors. The search strategy, refined in consultation with a research librarian, is detailed in Table 1.

**Table 1:** Search strategy. Database: Ovid MEDLINE(R) and Epub ahead of print, In-Process, In-Data-Review & other Non-Indexed citations and daily < 1946 to February 02, 2024. Adapted for EMBASE, IEEE, CINAHL, PsycINFO, Scopus, ERIC, web of science: core collection & compendex

#	Query
1	exp Artificial Intelligence/
2	(artificial intelligence or “natural language” or “deep learning” or “neural network*” or “support vector machine” or svm or “machine learning”).ti, ab, kw, jn.
3	Education, Medical, Graduate/ or “Internship and Residency”/
4	(“education, residency” or “residency educat*” or “residency train*” or “residential education” or “house staff” or “graduate medical educat*”).ti, ab, kw.
5	1 or 2
6	3 or 4
7	5 and 6

Studies were included if they [1] were original, peer-reviewed research articles; [2] involved participants in GME; [3] examined or evaluated artificial intelligence (AI) technologies—including machine learning, natural language processing, or large language models (LLMs) and [4] reported outcomes related to education, assessment, recruitment, or clinical training.

During the full-text review stage, studies were excluded if they were duplicates, did not report relevant outcomes, were categorized as gray literature such as editorials, opinion pieces or commentaries, lacked a clear AI intervention, were not related to GME populations or settings, or if the full text was unavailable. These exclusions ensured that included studies aligned with the scope and objectives of the review.

After the initial pilot screening, we employed a parallel dual review process for study selection, extraction, and analysis. Two independent reviewers (BGV, SC, TB, and CI) screened the titles, abstracts, and full texts, resolving disagreements with a third reviewer (RS). We used the Covidence platform for initial screening and deduplication. One reviewer (BGV) performed the data extraction, verified by a second reviewer (SC, TB, or CI) for accuracy and consistency. All the authors reviewed and reached a consensus on the final articles for data extraction.

#### Categorization and analysis

We employed a descriptive analysis approach to synthesize the study findings, categorizing studies based on AI applications, perceptions, and challenges in GME. These categories were developed through an iterative thematic coding process, where key themes were identified based on study objectives, methodologies, and reported outcomes. This approach ensured a comprehensive and applicable review.

## Results

### Overview

**Fig. 1:**
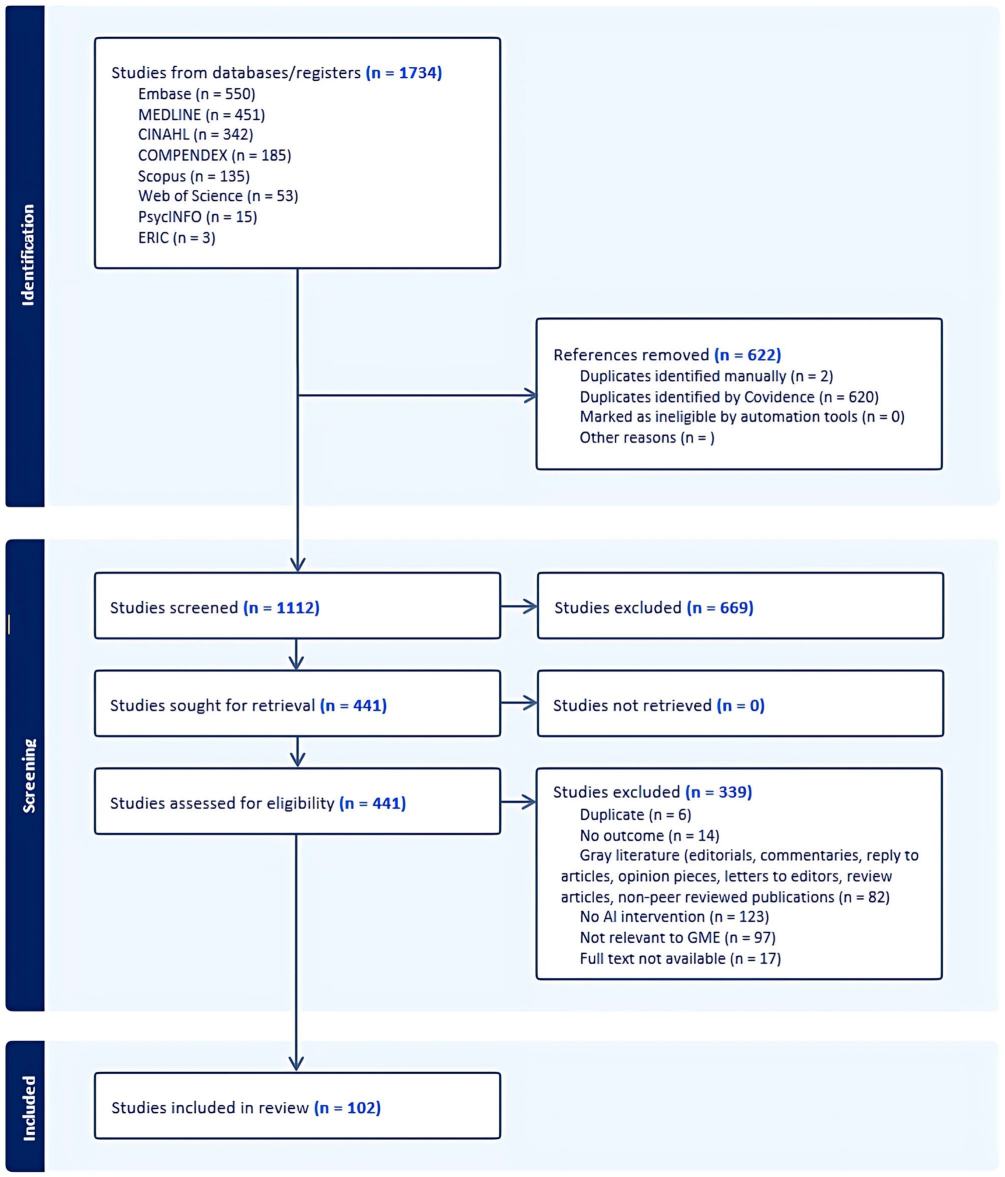
Preferred Reporting Items for Systematic Reviews and Meta-Analysis (PRISMA) flow chart

The PRISMA diagram in Fig. 1 shows the screening process. After removing duplicates from the 1,734 database citations, 1,112 records remained. After title and abstract screening, 441 articles were eligible for full-text evaluation. Of these, 102 were included in the final scoping review for data extraction.

**Fig. 2:**
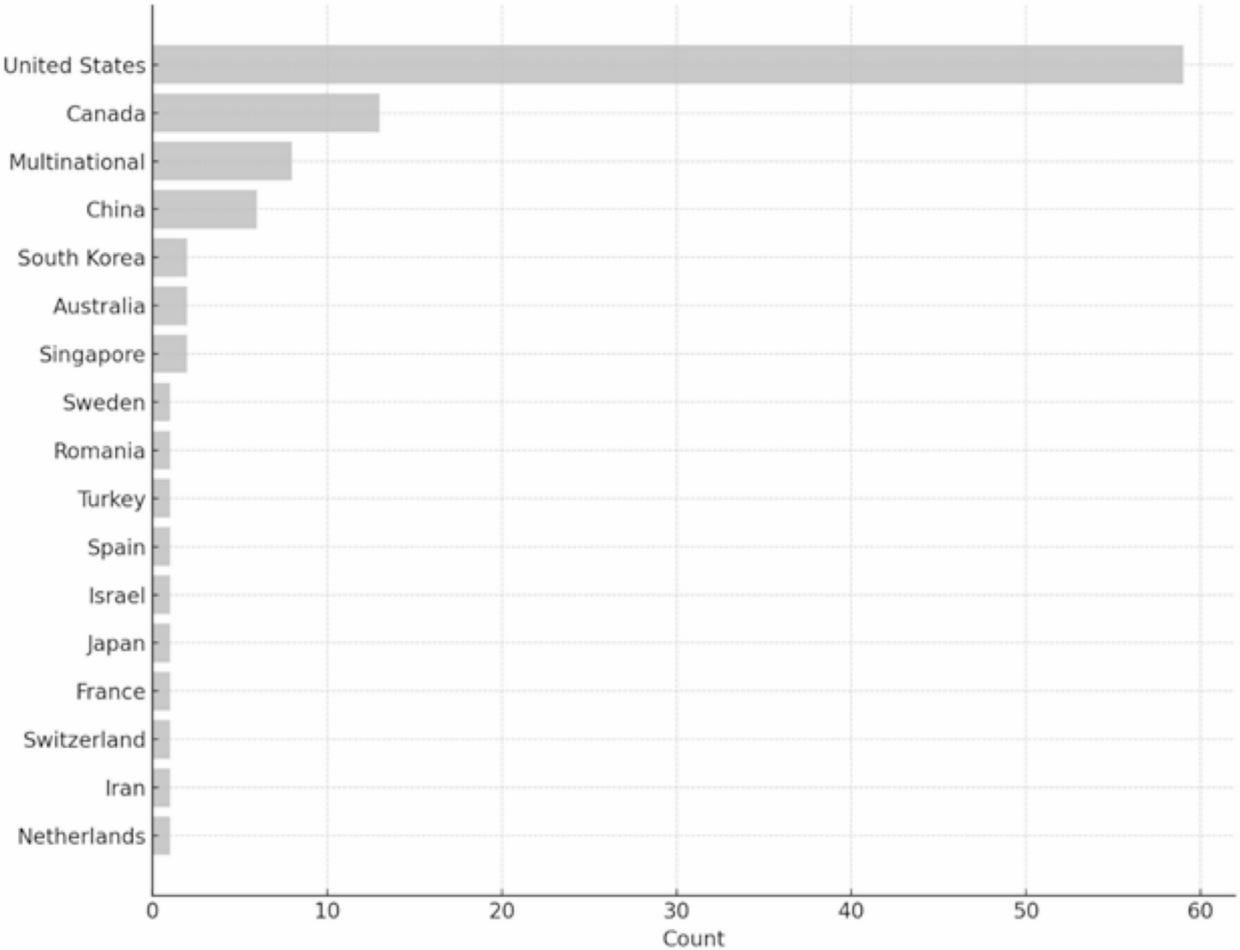
Study breakdown based on country where it was done

Figure 2 details the included studies conducted across 16 countries, with eight studies involving three or more countries. The origins include North America [72], Asia [14], multiple regions [8], Europe [6], and Oceania [2].

Most studies were published between 1997 and 2024, with the highest number published in 2023.

Table 2**(end of manuscript)** presents a detailed breakdown of the studies included. Radiology had the most publications [21], followed by general surgery [11] and emergency medicine [8] 37 studies involved multiple participant types, 34 studies included residents and chief residents, 14 studies had no human participants, 10 studies focused solely on applicants, 6 studies involved faculty and program directors (PDs), and 1 study included only medical students.

**Table 2:** Study description with summarized key results

Study Details (First author Year; Title; Country)	Aim/ Objective of the Study	Study Design	Study Characteristics (Specialty; Number; Participants)	AI Summary (Type of AI mentioned in the study; Study setting; AI being compared to)	Key AI-Related Results Summarized
Abbott [110] 2021; Natural Language Processing to Estimate Clinical Competency Committee Ratings; United States	Examine whether NLP can be used to estimate CCC ratings.	Predictive Study	General Surgery; 24 Residents	Natural Language Processing; Real world; AI vs. Human performance; AI vs. other AI tools	Combining NLP and non-NLP predictors (AUC = 0.87) outperforms using either non-NLP predictors (AUC = 0.84) or NLP predictors (AUC = 0.83) alone, showing enhanced model performance. Integrating both types of data improves predictive accuracy.
Ali [76] 2023; Performance of ChatGPT, GPT-4, and Google Bard on a Neurosurgery Oral Boards Preparation Question Bank; United States	To assess performance of three LLMs (GPT-3.5, GPT-4, and Google Bard) on a question bank designed specifically for neurosurgery oral boards examination preparation.	Non-Randomized Experimental Study	Neurosurgery; No human participants	Large Language Models; Theoretical; AI vs. other AI tools	GPT-4 outperformed GPT-3.5 and Bard in answering higher-order questions and imaging questions, with fewer hallucinations, demonstrating superior accuracy and reliability (all *P*-values < 0.05).
Alkadri [31] 2021; Utilizing a multilayer perceptron artificial neural network to assess a virtual reality surgical procedure; Canada	To demonstrate the benefits of artificial neural network algorithms in assessing and analyzing virtual surgical performances, focusing on a simulated annulus incision task during an anterior cervical discectomy and fusion scenario.	Development & Validation Study	Neurosurgery; Orthopedics; 23 Residents; Fellows	Artificial Neural Network; Simulations; No comparator	The artificial neural network model demonstrated an 80% testing accuracy when trained on nine selected surgical metrics, indicating reliable predictive performance across the evaluated categories.
Amirhajlou [43] 2019; Application of data mining techniques for predicting residents’ performance on pre-board examinations: A case study.; Iran	To predict the performance of residents on pre-board examinations based on the results of in-training examinations (ITE) using various educational data mining (DM) techniques	Predictive Study	Multiple; 841 Residents	Artificial Neural Network; Other: SVM, Linear regression; Real world; AI vs. other AI tools	ITE scores for PGY-2, PGY-3, and specialty training type predicted preboard examination scores, with MLP-ANN achieving the best performance and lowest error metrics (RMSE and MAE)
Andrews [105] 2021; Gender bias in resident evaluations: Natural language processing and competency evaluation; United States	To examine the differences in word use, competency themes, and length within written evaluations of internal medicine residents, considering the impact of both faculty and resident gender.	Observation Study	Medicine; Radiology; 413 Faculty	Natural Language Processing; Real world; No comparator	NLP was used to analyze 3,864 evaluations of internal medicine residents to assess sentiment and core competencies, and found no significant gender differences in the quantity or quality of feedback, despite female evaluators writing longer evaluations.
Anh [37] 2020; Towards near real-time assessment of surgical skills: A comparison of feature extraction techniques.; Australia	To compare different feature extraction techniques for automated surgical skill assessment in near real-time using motion analysis data.	Observation Study	General Surgery; 11 Residents; Faculty	Machine Learning; Other: Convolutional Neural Networks (CNN), Long Short Term Memory (LSTM), Principal Component Analysis (PCA), Discrete Fourier Transform (DFT), Discrete Cosine Transform (DCT), autoencoder, and others; Simulations; AI vs. other AI tools	Deep CNN achieved the highest classification accuracy for surgical tasks (96.84% suturing, 92.75% knot tying, 95.36% needle passing), while PCA also performed strongly (95.63% suturing, 90.17% knot tying, 90.26% needle passing
Ariaeinejad [42] 2020; A performance predictive model for emergency medicine residents; Canada	To develop a machine learning algorithm to identify patterns in resident performance and early identification of residents at risk.	Development & Validation Study	Emergency Medicine; not specified; Residents	Artificial Neural Network, Support Vector Machines (SVM), k-Nearest Neighbor (kNN); Real world; AI vs. other AI tools	The SVM model showed the highest accuracy in identifying at-risk residents with a sensitivity of 0.54, specificity of 0.74, and an AUC of 0.64
Ötleş [111] 2021; Using Natural Language Processing to Automatically Assess Feedback Quality: Findings From 3 Surgical Residencies; United States	To evaluate which NLP techniques best classify the quality of surgical trainee formative feedback recorded as part of a workplace assessment.	Non-Randomized Experimental Study	General Surgery; No human participant	Natural Language Processing; Real world; AI vs. other AI tools	The SVM NLP model achieved the highest mean accuracy for classifying feedback quality, with 0.64 for 4-category classification and 0.83 for binary classification, demonstrating the model’s capability to automate feedback quality assessment.
Baloul [32] 2022; Video Commentary & Machine Learning: Tell Me What You See, I Tell You Who You Are.; USA, Saudi Arabia	To apply Machine Learning (ML) in the context of a structured Video Commentary (VC) assessment to predict surgical residents training levels.	Predictive Study	General Surgery; 81 Residents	Machine Learning; Simulations; AI vs. Standard methods	Individual VC clip scores strongly correlated with PGY level (*p* = 0.001), and using a supervised machine learning model to predict PGY levels improved accuracy by 40% over traditional statistical analysis.
Bartoli [77] 2024; Probing artificial intelligence in neurosurgical training: ChatGPT takes a neurosurgical residents written exam; Switzerland	To assess how ChatGPT performs in generating and answering questions for a neurosurgical residents’ written exam.	Observation Study	Neurosurgery; 10 Residents	Large Language Models; Simulations; AI vs. Human performance	ChatGPT ranked 6th out of 11 participants, scoring 25.4 out of 42 questions.
Bissonnette [35] 2019; Artificial Intelligence Distinguishes Surgical Training Levels in a Virtual Reality Spinal Task.; Canada	To evaluate the potential of artificial intelligence (AI) as an assessment tool in virtual reality spine surgery simulation. Specifically, to determine if AI can uncover novel metrics of surgical performance and distinguish between senior and junior participants performing a virtual reality hemilaminectomy.	Observation Study	Neurosurgery; Orthopedics; 41 Residents; Fellows; Faculty; Medical Students	Machine Learning; Other: Machine Learning (Support Vector Machine, k-Nearest Neighbors, Linear Discriminant Analysis, Naive Bayes, Decision Tree); Simulations; AI vs. other AI tools	SVM achieved the highest accuracy (97.6%) in differentiating between senior and junior participants in a virtual reality spinal task, outperforming kNN (92.7%), LDA (87.8%), Decision Tree (70.7%), and Naive Bayes (65.9%)
Bond [10] 2019; Virtual Standardized Patient Simulation: Case Development and Pilot Application to High-Value Care.; United States	To develop virtual standardized patient (VSP) cases and provide preliminary evidence supporting their ability to provide experiential learning in high-value care (HVC).	Development & Validation Study	Medicine; Med-Peds; 14 Residents	Natural Language Processing; Simulations; AI vs. Human performance	In the study context, faculty and platform ratings of learner success in history-taking correlated strongly (ρ = 0.80, *P* = 0.02), with high interrater reliability (0.87). Learners ordered a median of two unnecessary tests, correctly diagnosed within the top three 82% of the time, and completed 56% of optimal treatments.
Boolchandani [53] 2023; Words Used in Letters of Recommendation for Pediatric Residency Applicants: Demographic Differences and Impact on Interviews.; United States	To describe differences in agentic (achievement) and communal (relationship) terms in LORs for pediatric residency candidates by applicant and letter writer demographics and to examine if LOR language is associated with interview statusâ€‹â€‹.	Observation Study	Pediatrics; 573 Residency applicants	Natural Language Processing; Real world; No comparator	NLP was used to analyze 2,094 pediatric residency LORs. 53% of LORs were agency biased, 25% communal biased, and 23% neutral. There were no significant differences in LOR language based on the applicant’s gender or race, but male letter writers used significantly more agentic terms than female writers. Neutral-toned LORs were more likely associated with interview invitations
Booth [112] 2023; Competency-Based Assessments: Leveraging Artificial Intelligence to Predict Subcompetency Content; United States	To develop and evaluate a Natural Language Processing (NLP) algorithm that automatically categorizes narrative feedback into corresponding Accreditation Council for Graduate Medical Education Milestone 2.0 subcompetenciesâ€‹â€‹.	Development & Validation Study	Anesthesiology; 376 Residents; Faculty	Natural Language Processing; Real world; No comparator	The model performed well for professionalism, interpersonal and communication skills, and practice-based learning (AUC 0.79, 0.79, and 0.75), with fair to excellent results for medical knowledge and patient care (AUC 0.66–0.84 and 0.63–0.88), but poorly for systems-based practice (AUC 0.59). It also provided quick, organized feedback for trainees via a web-based application.
Brown [113] 2024; Evaluating the Impact of Assessment Metrics for Simulated Central Venous Catheterization Training.; United States	To examine the effectiveness of the DHRT in training residents on needle tip tracking and aspiration skills.	Observation Study	Multiple; 163 Residents	Decision support systems; Simulations; AI vs. Standard methods	Tip tracking rates above 40% were 2.3 times more likely to result in successful venous access than below 10%, and aspiration rates above 80% were 2.6 times more likely to result in success than below 10%. Proper techniques reduced complications, with resident performance improving in all metrics except tip tracking
Brunye [106] 2023; Machine learning classification of diagnostic accuracy in pathologists interpreting breast biopsies; United States	To explore the feasibility of using machine learning to predict accurate versus inaccurate diagnoses made by pathologists based on their spatiotemporal viewing behavior when evaluating digital breast biopsy images.	Predictive Study	Pathology; 140 Residents; Faculty	Machine Learning; Real world; AI vs. Human performance	Random Forest classifier achieved the best performance with a test accuracy of 0.81 and AUROC of 0.86. Attention distribution and focus on critical regions predicted diagnostic accuracy, with incremental improvements from case-level and pathologist-level information.
Burk-Rafel [58] 2021; Development and Validation of a Machine Learning-Based Decision Support Tool for Residency Applicant Screening and Review.; United States	To develop and validate a machine learning-based decision support tool (DST) for residency applicant screening and review.	Development & Validation Study	Medicine; 8243 Residency applicants	Machine Learning; Real world; AI vs. Human performance	The Random Forest classifier showed high performance (AUROC 0.95, AUPRC 0.76, sensitivity 91%, specificity 85%). Removing USMLE scores slightly reduced performance (AUROC 0.94, AUPRC 0.72). The DST identified 20 initially overlooked applicants for interviews.
Chassagnon [98] 2023; Learning from the machine: AI assistance is not an effective learning tool for resident education in chest x-ray interpretation; France	To assess whether a computer-aided detection (CADe) system could serve as a learning tool for radiology residents in chest X-ray (CXR) interpretation.	Observation Study	Radiology; 8 Residents	Deep Learning; Real world; AI vs. Human performance	The CADe system improved residents’ sensitivity (43–53%), specificity (90–94%), and accuracy (81–86%) during use, but performance returned to baseline post-intervention, indicating temporary enhancement.
Chen [74] 2019; Developing a More Responsive Radiology Resident Dashboard; United States	To develop and evaluate a radiology resident dashboard (Trove) that uses NLP and machine learning to track and visualize resident caseloads, providing insights into their training progress and identifying gaps.	Development & Validation Study	Radiology; No human participants	Machine Learning; Natural Language Processing; Other: Deep Learning; Real world; No comparator	CNNs achieved 96.4% accuracy for large datasets across various ICD codes, while GRUs achieved an even higher accuracy of 97.0%, demonstrating high model performance.
Chen [23] 2023; Radiology Residents Perceptions of Artificial Intelligence: Nationwide Cross-Sectional Survey Study; China	Radiology residents perception of AI	Survey study	Radiology; 3666 Residents	General AI; Machine Learning; Real world; No comparator	74% of respondents see AI as non-threatening and beneficial to workflow, while 95% of residents want AI education, yet 20% of programs lack formal AI training.
Cheung [86] 2023; ChatGPT versus human in generating medical graduate exam multiple choice questions-A multinational prospective study (Hong Kong S.A.R., Singapore, Ireland, and the United Kingdom).; Multinational	To assess the quality of MCQs produced by ChatGPT for graduate medical examinations compared to questions written by university professoriate staff.	Survey study	General; 7 Faculty	Large Language Models; Real world; AI vs. Human performance	AI-generated MCQs matched human-created ones in quality across most domains but were slightly inferior in relevance. ChatGPT notably produced MCQs much faster than humans.
Cohen [81] 2023; Performance of ChatGPT in Israeli Hebrew OBGYN national residency examinations.; Israel	To evaluate the performance of ChatGPT in Hebrew OBGYN (Phase 1) examination and compare it to the performance of real-life OBGYN residents and ChatGPT’s performance in English medical exams.	Non-Randomized Experimental Study	ObGyn; not specified Residents	Large Language Models; Real world; AI vs. Human performance	ChatGPT scored 38.7% on Hebrew OBGYN questions, significantly lower than residents’ 68.4% (*p* < 0.001), and performed better on English tests with 60.7% accuracy compared to 38.7% in Hebrew (*p* < 0.001)
Collado-Mesa [19] 2018; The Role of Artificial Intelligence in Diagnostic Radiology: A Survey at a Single Radiology Residency Training Program; United States	To establish a baseline understanding of the awareness and perceptions of AI among radiology trainees and attending radiologists.	Survey study	Radiology; 69 Residents; Fellows; Faculty	General AI; Real world; No comparator	36% of participants hadn’t read an AI article in the past year, 29% used AI tools daily, and trainees were more concerned about AI’s impact on future job roles compared to attending radiologists.
Dimitroyannis [104] 2023; Residency Education Practices in Endoscopic Skull Base Surgery; United States	To analyze endoscopic skull base surgery education methods by surveying NASBS members.	Survey study	Neurosurgery, Otolaryngology; 60 Faculty	General AI; Real world; No comparator	Standardization and use of simulation, AI, and VR should be at the forefront of educational practices in the next 5–10 years.
DiPietro [107] 2019; Segmenting and classifying activities in robot-assisted surgery with recurrent neural networks.; United States	To improve automated segmentation and classification of surgical activities in robot-assisted surgery using RNNs	Observation Study	General; 15 subjects for MISTIC-SL dataset; 8 subjects for JIGSAWS dataset Residents; Medical Students	Recurrent Neural Networks (RNNs); Simulations; AI vs. Standard methods	This study utilized recurrent neural networks (RNNs) to segment and classify activities in robot-assisted surgery, comparing four RNN architectures. The models achieved state-of-the-art performance, with the GRU architecture yielding the lowest error rate of 8.6% and normalized edit distance of 9.3% for maneuver recognition. The results demonstrated that RNNs could effectively recognize both gestures and higher-level maneuvers, providing a foundation for automated, targeted assessment and feedback in surgical training.
Drum [57] 2023; Using Natural Language Processing and Machine Learning to Identify Internal Medicine-Pediatrics Residency Values in Applications.; United States	To use NLP and ML to identify values associated with resident success in internal medicine-pediatrics residency applications	Observation Study	Internal Med-Pediatrics; 185 Residency applicants	Machine Learning; Natural Language Processing; Real world; AI vs. Human performance	MLM showed moderate sensitivity (0.64), high specificity (0.97), PPV (0.64), NPV (0.97), and an F1 score of 0.63 in identifying values from unstructured narrative data, with the mean number of annotations per application significantly correlating with interview invitation status.
Ebina [114] 2022; Objective evaluation of laparoscopic surgical skills in wet lab training based on motion analysis and machine learning.; Japan	To build a skill assessment system providing objective feedback to trainees based on motion metrics of laparoscopic surgical instruments	Development & Validation Study	Multiple; 70 Residents; Faculty; Medical Students	Machine Learning; Simulations; No comparator	Speed-related parameters positively correlated with mean GOALS scores, while efficiency-related parameters negatively correlated. SVR had the highest accuracy in the tissue dissection task (MAEmedian = 2.2352), and PCA-SVR in the parenchymal-suturing task (MAEmedian = 1.2714)
El Saadawi [70] 2008; A natural language intelligent tutoring system for training pathologists: implementation and evaluation.; United States	To develop and evaluate a Natural Language Interface (NLI) for an Intelligent Tutoring System (ITS) in Diagnostic Pathology, focusing on teaching residents to examine pathologic slides and write accurate pathology reports while providing immediate feedback on errors.	Development & Validation Study	Pathology; 20 Residents	Natural Language Processing; Simulations; No comparator	NLP system showed a precision of 0.90 and a recall of 0.84. Report writing skills significantly improved after one tutoring session, with a four-fold increase in learning gains, unaffected by the timing of feedback.
Fang [96] 2022; Artificial intelligence-based pathologic myopia identification system in the ophthalmology residency training program; China	To evaluate the effectiveness of the AI-based pathologic myopia (PM) identification system in the ophthalmology residency training program and assess the residentsâ€™ feedback on this system.	Randomized controlled trial	Ophthalmology; 90 Residents	Decision support systems; Real world; AI vs. Human performance	Post-lecture scores significantly improved in the AI group (*p* < 0.0001) compared to both traditional groups, which showed no significant improvement (*p* = 0.302 and *p* = 0.158). Residents reported high satisfaction with the AI system, noting its effectiveness in enhancing their learning and understanding of PM
Feng [92] 2023; Deep Neural Network Augments Performance of Junior Residents in Diagnosing COVID-19 Pneumonia on Chest Radiographs; Singapore	To assess the effectiveness of a deep neural network in distinguishing COVID-19 from other types of pneumonia, and to determine its potential contribution to improving the diagnostic precision of junior residents.	Non-Randomized Experimental Study	Radiology; 3 Residents	Deep neural network; Real world; No comparator	The AI model achieved an AUC of 0.9520 on the internal test set and 0.8588 on the external test set. With AI assistance, junior residents showed significant improvements in diagnostic accuracy: JR1’s sensitivity for COVID-19 pneumonia increased from 0.3889 to 0.6250, and specificity for non-pneumonia from 0.9091 to 0.9339. Similarly, JR2’s specificity for non-pneumonia improved from 0.9008 to 0.9835.
Gao [102] 2019; Constructing a Chinese electronic medical record corpus for named entity recognition on resident admit notes.; China	To construct a Chinese electronic medical record corpus for named entity recognition on resident admit notes and to evaluate the effectiveness of an end-to-end deep neural network model for medical named entity recognition (NER).	Development & Validation Study	General; 255 notes Resident	Deep neural network; Real world; No comparator	The annotation scheme and the model for NER in this paper are effective to extract medical named entity from RANs and provide the basis for fully excavating the patients information.
Gates [115] 2023; Association of Gender and Operative Feedback Quality in Surgical Residents.; United States	To explore the quality of narrative feedback among trainee-faculty gender dyads in an operative workplace-based assessment (WBA).	Observation Study	General Surgery; 2319 Residents	Natural Language Processing; Real world; No comparator	NLP analyzed 67,434 operative performance evaluations for gender-based differences in feedback quality among surgical residents. Male faculty provided more narrative feedback than female faculty. Female residents received higher quality feedback compared to male residents, but no significant differences were found based on faculty-resident gender dyad.
Gong [101] 2021; Characterizing styles of clinical note production and relationship to clinical work hours among first-year residents.; United States	To characterize variation in clinical documentation production patterns and their relationship to work hours	Observation Study	Medicine; Residents; 50	Machine Learning; Real world; No comparator	Unsupervised machine learning analyzed clinical note production among 50 first-year residents, identifying 10 note-level and 5 user-level patterns. Residents writing in dispersed sessions had higher median daily work hours, while single-session writers had lower hours.
Gray [56] 2023; Examining Implicit Bias Differences in Pediatric Surgical Fellowship Letters of Recommendation Using Natural Language Processing.; United States	To analyze the prevalence and type of bias in letters of recommendation (LOR) for pediatric surgical fellowship applications from 2016–2021 using natural language processing (NLP).	Observation Study	Pediatric surgery; 182 Residency applicants	Natural Language Processing; Real world; No comparator	NLP analyzed bias in 701 pediatric surgical fellowship LORs from 182 applicants (2016–2021). Results showed Black applicants had the highest mean polarity, while Hispanic applicants had the lowest. Significant differences in “anger” intensity were found among Black, Asian, and Hispanic applicants. NLP effectively identified subtle biases, impacting fellowship matching.
Gupta [78] 2023; Applying GPT-4 to the Plastic Surgery Inservice Training Examination; United States	To determine if GPT-4 could be exploited as an instrument for plastic surgery graduate medical education by evaluating its performance on the Plastic Surgery Inservice Training Examination (PSITE).	Observation Study	Plastic surgery; No human participant	Large Language Models; Simulations; No comparator	GPT-4 achieved 77.3% accuracy on the PSITE exam, using logical reasoning in 95.0% of cases, internal information in 98.3%, and external information in 97.5%. A significant difference in logical reasoning usage was noted between correct and incorrect answers.
Han [91] 2020; Augmented Intelligence Dermatology: Deep Neural Networks Empower Medical Professionals in Diagnosing Skin Cancer and Predicting Treatment Options for 134 Skin Disorders.; South Korea	The performance of CNN needs to be tested in an environment similar to real practice, which requires dis- tinguishing skin cancer from numerous other skin disorders including inflammatory and infectious conditions. In addi- tion, the robustness and repeatability of this approach require further validations	Predictive Study	Dermatology; 47 Residents; Faculty	Convolutional Neural Networks; Simulations; No comparator	The algorithm improved clinicians’ malignancy prediction sensitivity by 12.1% and specificity by 1.1% (both *P* < 0.0001), and increased non-medical professionals’ sensitivity by 83.8% (*P* < 0.0001). It also enhanced top-1 and top-3 accuracies for four doctors in classifying 134 diseases.
Hernandez-Rodriguez [116] 2023; Development and validation of an educational software based in artificial neural networks for training in radiology (JORCAD) through an interactive learning activity.; Spain	To validate an educational software tool (JORCAD) for training residents in Radiology	Development & Validation Study	Radiology; 26 Residents; Faculty	CNN; Real world; No comparator	JORCAD had high mean ratings for software tools (4.12–4.77), significant learning utility (4.54–4.77), and strong agreement among participants on the software’s effectiveness for training in thorax CT and mammography cases. The CAD tool was particularly effective in reinforcing diagnostic confidence and enhancing educational experiences​
Holden [39] 2019; Machine learning methods for automated technical skills assessment with instructional feedback in ultrasound-guided interventions.; Canada	Develop and validate skills assessment methods in ultrasound-guided interventions that are transparent and configurable	Development & Validation Study	General; 24 Residents; Faculty	Other: Decision Trees, Fuzzy Inference Systems; Simulations; AI vs. Human performance; AI vs. Standard methods	Decision Trees had median errors of 1.7 in-plane and 1.5 out-of-plane, while Fuzzy Inference Systems had errors of 1.8 in-plane and 3.0 out-of-plane, performing comparably to state-of-the-art methods and providing useful feedback.
Holmes [80] 2023; Evaluating Large Language Models in Ophthalmology; China, USA	Evaluate and compare the performance of three LLMs in answering ophthalmology questions	Non-Randomized Experimental Study	Ophthalmology; 18 Residents; Faculty; Medical Students	Large Language Models; Simulations; AI vs. Human performance; AI vs. other AI tools	GPT-4 performed at a level comparable to attending physicians, while GPT-3.5 and PaLM2 were slightly below the master’s level. GPT-4 also demonstrated higher stability (mean correlation 0.83) and confidence (45% accuracy) compared to GPT-3.5 and PaLM2. All LLMs, outperformed medical undergraduates in general assessments.
Homayounieh [90] 2021; An Artificial Intelligence-Based Chest X-ray Model on Human Nodule Detection Accuracy from a Multicenter Study; USA, Germany	To assess if a novel AI algorithm can help detect pulmonary nodules on radiographs at different levels of detection difficulty	Non-Randomized Experimental Study	Radiology; 9 Residents & Faculty	Machine Learning; Real world; AI vs. Human performance	AI-aided interpretation improved mean detection accuracy by 6.4% (95% CI, 2.3–10.6%) and partial AUCs by 5.6% (95% CI, − 1.4–12.0%). Junior radiologists showed greater sensitivity improvement (12% vs. 9%) with AI aid, while senior radiologists had similar specificity improvement (4%).
Huisman [22] 2021; An international survey on AI in radiology in 1,041 radiologists and radiology residents part 1: fear of replacement, knowledge, and attitude.; Multinational	Investigate the knowledge and attitude towards AI among radiologists and residents, focusing on fear of replacement by AI and positive attitudes towards AI .	Survey study	Radiology; Residents; Faculty; 1041	General AI; Deep learning; Real world; No comparator	Limited AI knowledge is linked to a fear of being replaced, whereas intermediate to advanced knowledge corresponds with positive attitudes. This fear is more common among males and those with basic AI understanding. Conversely, positive attitudes are typically found in younger individuals, males, those with scientific backgrounds, and active professional social media users.
Humar [79] 2023; ChatGPT Is Equivalent to First-Year Plastic Surgery Residents: Evaluation of ChatGPT on the Plastic Surgery In-Service Examination; United States	Evaluate the performance of ChatGPT on the Plastic Surgery In-Service Examination and compare it to residents’ performance nationallyâ€‹â€‹.	Observation Study	Plastic surgery; No human participants	Large Language Models; Real world; AI vs. Human performance	ChatGPT answered 55.8% of exam questions correctly, performing on par with a first-year integrated plastic surgery resident but poorly compared to more advanced residents.
Jalali [117] 2017; LiveBook: Competence Assessment with Virtual-Patient Simulations; Canada	To present and evaluate the LiveBook interactive simulation system for competence assessment with virtual-patient simulations	Development & Validation Study	Pediatrics; 23 Residents	Natural Language Processing; Decision support systems; Simulations; No comparator	The article discusses the creation and ongoing validation process of Livebook, noting that no actual study results have been completed at the time of publication.
Johnstone [64] 2023; Artificial intelligence software can generate residency application personal statements that program directors find acceptable and difficult to distinguish from applicant compositions; United States	To determine if AI-generated personal statements for anesthesiology residency applications are acceptable to program directors	Non-Randomized Experimental Study	Anesthesiology; 94 Residency program directors	Natural Language Processing; Real world; No comparator	61% of program directors did not identify AI-generated statements related to athletics, and 80% failed to detect those related to cooking. Overall, AI-generated statements were rated as acceptable and were challenging to distinguish from those written by humans
Kelahan [71] 2016; Call Case Dashboard: Tracking R1 Exposure to High-Acuity Cases Using Natural Language Processing.; United States	To track first-year radiology residents’ exposure to high-acuity cases using Natural Language Processing (NLP).	Observation Study	Radiology; 4 Residents	Natural Language Processing; Real world; No comparator	NLP achieved a specificity of 95-100% and a sensitivity of 92.3-100% for various conditions, accurately tracking high-acuity cases and identifying educational deficiencies in resident training.
Kennedy [21] 2022; Canadian Oncology Residents Knowledge of and Attitudes Towards Artificial Intelligence and Machine Learning; Canada, Brazil	Examine perceptions and knowledge of Canadian oncology residents and fellows with respect to AI technologies	Survey study	Radiation Oncology, Medical Oncology; 57 Residents; Fellows	General AI; Real world; No comparator	67% of residents recognized the importance of AI education, and 70% wanted to learn more, yet 88% lacked formal AI education. Additionally, 98% believed AI could replace some to all medical activities in radiation oncology, and 63% felt limited knowledge in math and programming hindered their understanding of AI.
KocerTulgar [27] 2023; Anesthesiologists’ Perspective on the Use of Artificial Intelligence in Ultrasound-Guided Regional Anaesthesia in Terms of Medical Ethics and Medical Education: A Survey Study.; Turkey	Determine how anesthesiology and reanimation specialists in Turkey perceive the use of AI in ultrasound-guided regional anesthetic applications in terms of medical ethics and education, as well as their perspectives on potential ethical issues.	Survey study	Anesthesiology; 285 Faculty	Machine Learning; Real world; No comparator	80% of respondents believed AI would benefit patients, 86.7% saw advantages for resident training, 81.4% for post-graduate medical education, and 80.7% thought it would reduce practice complications. Ethically, 78.25% were fine with anonymous sonographic data capture, but 67% had concerns about accountability for AI inaccuracies.
Kumar [41] 2012; Objective measures for longitudinal assessment of robotic surgery training.; United States	Development and validation of a novel automated and objective framework for the assessment of training in robotic surgery	Development & Validation Study	Cardiothoracic surgery; 12 Residents; Fellows; Faculty	Other: Automated motion recognition system; Real world; Simulations; No comparator; AI vs. Human performance	The system could objectively differentiate between operational and technical skills. Over time, trainees’ skill parameters converged toward those of expert surgeons. The study reported significant improvements in task completion times and motion efficiency, with high accuracy in classifying expert versus non-expert performance (suturing task: 83.33%, manipulation task: 76.25%
Lee [97] 2020; Application of deep learning to the diagnosis of cervical lymph node metastasis from thyroid cancer with CT: external validation and clinical utility for resident training; South Korea	To validate a deep learning model’s diagnostic performance for CT diagnosis of cervical LNM from thyroid cancer and evaluate its clinical utility for resident training.	Development & Validation Study	Radiology; 7 Residents; Faculty	Other: CNN; Real world; AI vs. other AI tools	The overall AUROC of the eight deep learning algorithms was 0.846, with Xception being the best-performing model (AUROC 0.884). Its diagnostic accuracy, sensitivity, specificity, positive predictive value, and negative predictive value were 82.8%, 80.2%, 83.0%, 83.0%, and 80.2%, respectively. Introducing the CAD system provided more assistance to underperforming trainees (*p* = 0.046) and significantly increased overall confidence levels from 3.90 to 4.30 (*p* < 0.001).
Lin [72] 2014; A content-boosted collaborative filtering algorithm for personalized training in interpretation of radiological imaging.; China, Canada	Develop a personalized training program in radiology education using a content-boosted collaborative filtering (CBCF) algorithm	Development & Validation Study	Radiology; 15 Residents	Other: Content-Based Filtering (CBF), Collaborative Filtering (CF); Real world; AI vs. other AI tools	CBCF outperformed pure CBF and CF methods by 13.33% and 12.17% in prediction precision, respectively, indicating its potential for developing personalized training systems in radiology education.
Lui [52] 2018; Tracking resident cognitive maturation with natural language processing; United States	To develop and test software for tracking the cognitive maturation of medical residents using NLP to analyze freetext evaluations.	Development & Validation Study	Emergency Medicine; 100 Residents	Natural Language Processing; Simulations; No comparator	The algorithm correctly identified 22 out of 25 notes where the “laggard” archetype predominated, demonstrating its accuracy. Feedback mechanisms were validated through the significant correlation between the identified linguistic markers and the developmental progression noted by faculty, supporting the system’s effectiveness in real-time tracking and tailored educational interventions
Lum [83] 2023; Can Artificial Intelligence Pass the American Board of Orthopaedic Surgery? An Analysis of 3900 Questions; United States	To determine if ChatGPT can pass the American Board of Orthopaedic Surgery Examination	Non-Randomized Experimental Study	Orthopaedics; No human participant	Large Language Models; Real world; AI vs. Human performance	ChatGPT correctly answered 47% of OITE questions, with accuracy decreasing as question complexity increased. It performed at the 40th percentile for PGY1, 8th percentile for PGY2, and 1st percentile for PGY3-PGY5, not meeting the 10th percentile passing threshold for PGY5s on the ABOS exam. By question taxonomy, it scored 54% on recognition and recall, 51% on comprehension and interpretation, and 34% on application of knowledge questions.
Madhavan [118] 2014; Evaluation of Documentation Patterns of Trainees and Supervising Physicians Using Data Mining.; United States	Evaluate documentation patterns of trainees and supervising physicians using data mining	Observation Study	Multiple; 26,802 notes Residents; Faculty	Natural Language Processing; Real world; No comparator	Most resident notes were entered in the afternoon (33%) and late morning (31%). Attending physicians provided teaching attestations within 24 h for 73% of records. Surgical residents placed notes more often before noon, while nonsurgical faculty typically attested within 24 h.
Mahajan [85] 2023; Assessment of Artificial Intelligence Performance on the Otolaryngology Residency In-Service Exam; United States	Determine the potential use and reliability of a large language model (ChatGPT) for answering otolaryngology in-service exam questions and assess its efficacy for surgical trainees.	Observation Study	Otolaryngology; No human participant	Large Language Models; Real world; No comparator	ChatGPT’s performance varied by question difficulty, with overall correct answer and explanation rates of 53% and 54%. For easy questions, the rates were 68% and 69%; for moderate questions, 52% and 53%; and for hard questions, 38% and 39%. Performance differences were statistically significant across difficulty levels.
Mahtani [63] 2023; A New Tool for Holistic Residency Application Review: Using Natural Language Processing of Applicant Experiences to Predict Interview Invitation.; United States	Develop an NLP tool to automate the review of residency applicantsâ€™ narrative experience entries and predict interview invitations.	Development & Validation Study	Medicine; 6403 Residency applicants	Natural Language Processing; Real world; No comparator	NLP-only model achieved an AUROC of 0.80 and AUPRC of 0.49, while the structured data-only model had superior performance with an AUROC of 0.92 and AUPRC of 0.74. Combining NLP and structured data, the model maintained an AUROC of 0.92 and AUPRC of 0.73. Terms indicating active leadership, research involvement, and efforts in social justice and health disparities positively correlated with interview invitations
Marchetti [94] 2020; Computer algorithms show potential for improving dermatologists’ accuracy to diagnose cutaneous melanoma: Results of the International Skin Imaging Collaboration 2017.; United States	Determine if computer algorithms from an international melanoma detection challenge can improve dermatologist melanoma diagnostic accuracy.	Observation Study	Dermatology; 17 Residents; Faculty	Deep learning, neural networks; Real world; AI vs. Human performance	The top computer algorithm outperformed dermatologists and residents in melanoma classification with an ROC area of 0.87, compared to 0.74 and 0.66, respectively (*p* < 0.001). Dermatologists’ sensitivity was 76.0%, while the algorithm’s specificity was higher at 85.0% versus 72.6% (*p* = 0.001). Imputing algorithm classifications for uncertain dermatologist evaluations increased sensitivity to 80.8% and specificity to 72.8%.
Marquis [25] 2023; Results of the 2020 Survey of the American Alliance of Academic Chief Residents in Radiology; United States	Summarize the 2020 A3CR2 chief resident survey, focusing on residency program practices, benefits, training choices, and perceptions on corporatization, NPPs, and AI in radiology.	Survey study	Radiology; 174 Chief residents	Machine Learning; General AI; Real world; No comparator	74% of respondents believe AI does not threaten the job market and could improve workflow efficiency. Despite this, 95% of residents expressed a need for AI and machine learning education, but 20% of programs currently do not offer formal AI education.
Merritt [68] 2022; Implementation and Evaluation of an Artificial Intelligence Driven Simulation to Improve Resident Communication With Primary Care Providers; United States	Implement and evaluate an AI-driven simulation to improve resident communication with primary care providers (PCPs).	Development & Validation Study	Pediatrics; 17 Residents	Other: IBM Watson’s integrated cognitive computing and linguistic modeling; Simulations; No comparator	94% of participants were fully engaged during the AI simulation. Most found it beneficial: 76.4% said it reinforced key communication elements, and 70.6% believed it would positively impact future communications with PCPs. The AI simulation was rated equally or more favorably than reading (76.5%), didactics (70.6%), and online education (88.3%), but less favorably than live simulated encounters (64.7%). Only 52.9% saw it as less effective than real-time observation.
Muntean [73] 2023; Artificial Intelligence for Personalised Ophthalmology Residency Training; Romania	To develop an AI framework for personalized case-based ophthalmology residency training	Development Study	Ophthalmology; 10 Residents	Deep learning; Real world; No comparator	Potential to standardize and personalize ophthalmology residency training
Neves [51] 2021; Using Machine Learning to Evaluate Attending Feedback on Resident Performance.; United States	Use machine learning to evaluate attending feedback on resident performance	Predictive Study	Anesthesiology; 146 Faculty	Machine Learning; Real world; No comparator	Models predicting feedback traits achieved 74.4-82.2% accuracy. Utility category predictions were 82.1% accurate, with 89.2% sensitivity and 89.8% precision for low-utility predictions. Quality category predictions were 78.5% accurate, with 86.1% sensitivity and 85.0% precision for low-quality predictions. The program processed data and generated predictions within minutes, compared to 15 h for manual scoring of 200 comments over two weeks.
Nori [75] 2023; Capabilities of GPT-4 on Medical Challenge Problems; United States	To investigate the capabilities of GPT-4 on medical challenge problems.	Non-Randomized Experimental Study	General; No human participant	Large Language Models; Theoretical; AI vs. other AI tools	GPT-4 surpasses the USMLE passing score by over 20 points, outperforming GPT-3.5 and Med-PaLM. It shows improved calibration, accurately predicting its correct answers’ likelihood. A case study also highlights GPT-4’s ability to explain medical reasoning, tailor explanations to students, and create interactive counterfactual scenarios.
Olsson [93] 2006; Decision support for the initial triage of patients with acute coronary syndromes.; Sweden	To develop an automated tool for ECG analysis for detecting transmural ischaemia and assess its impact on interns’ classifications	Development & Validation Study	Cardiology; 3 Residents	Artificial Neural Network; Real world; AI vs. Human performance	Three interns improved their sensitivity from 68–93% and specificity from 92–87% with neural network assistance. The neural network alone achieved 95% sensitivity and 88% specificity. The 23–26% sensitivity increase for all three interns was statistically significant (*P* < 0.001).
Ooi [24] 2021; Attitudes toward artificial intelligence in radiology with learner needs assessment within radiology residency programmes: a national multi-programme survey; Singapore	To assess attitudes and learner needs of radiology residents and faculty regarding AI/ML in radiology	Survey study	Radiology; 125 Residents; Faculty	Machine Learning; Other: General AI; Real world; No comparator	88.8% of respondents believe AI/ML will drastically change radiology, with 76% finding it exciting and 80% still choosing to specialize in it. While 64.8% consider themselves AI/ML novices, 76% want to improve their knowledge, and 67.2% are interested in AI/ML research. A majority (84.8%) think AI/ML should be included in residency curricula, but 59.2% report insufficient AI/ML education in their programs. Male and tech-savvy individuals show better technical understanding and engagement in AI/ML activities.
Oropesa [40] 2014; Supervised classification of psychomotor competence in minimally invasive surgery based on instruments motion analysis.; Netherlands	To compare three classification methods for assessing psychomotor skills in minimally invasive surgery (MIS).	Non-Randomized Experimental Study	General Surgery; 42 Residents; Faculty; Medical Students	Other: Support Vector Machines, Linear Discriminant Analysis, Adaptive Neuro-Fuzzy Inference Systems; Simulations; AI vs. other AI tools	The classifiers achieved mean accuracies of 71% (LDA), 78.2% (SVM), and 71.7% (ANFIS). No statistically significant differences were found between the classifiers, demonstrating that machine learning classifiers can effectively assess surgical competence, suggesting their integration into surgical training programs for objective evaluations.
Ortiz [62] 2023; Words matter: using natural language processing to predict neurosurgical residency match outcomes.; United States	To compare the performance of machine learning models trained on applicant NLORs and demographic data to predict neurosurgical residency match outcomes and investigate whether narrative language is predictive of SLOR rankings.	Predictive Study	Neurosurgery; 391 Residency applicants	Natural Language Processing; Real world; AI vs. Human performance	Both NLOR and demographic models predicted match outcomes with similar effectiveness (AUC values of 0.75 and 0.80, respectively). Words like “outstanding,” “seamlessly,” and “AOA” were predictive of match success. NLORs provided additional insights into applicant fit beyond demographic data.
Ouyang [103] 2024; Leveraging Historical Medical Records as a Proxy via Multimodal Modeling and Visualization to Enrich Medical Diagnostic Learning; China	Enhance the learning experience of interns and novice physicians in diagnostic skills through the use of ML models trained on historical medical data and multimodal modeling and visualization	Development & Validation Study	General; 5 Residents; Faculty	Others: Multimodal models including ClinicalBERT, ConvNeXt, XGBoost; Real world; No comparator	It achieved an accuracy of 91.6% (decision-level fusion) in diagnosing cervical spine disorders. The system was validated through two case studies and expert interviews, demonstrating improved learning outcomes for interns and novice physicians by providing visual diagnostics and comparative analysis of patient data
Patel [65] 2023; Distinguishing Authentic Voices in the Age of ChatGPT: Comparing AI-Generated and Applicant-Written Personal Statements for Plastic Surgery Residency Application.; United States	To explore whether residency application reviewers could discern ChatGPT-generated personal statements from those written by human applicants.	Non-Randomized Experimental Study	Plastic surgery; 10 Residents; Faculty	Large Language Models; Real world; AI vs. Human performance	There was no significant difference in ratings for readability, originality, authenticity, and overall quality between computer-generated and applicant essays (all *P* > 0.05). Although raters were less inclined to grant interviews to computer-generated essays, the difference was not significant (58% vs. 78%, *P* = 0.12).
Paul [95] 2023; Impact of an Artificial Intelligence Algorithm on Diabetic Retinopathy Grading by Ophthalmology Residents; United States	To determine whether AI significantly affects the performance of diabetic retinopathy (DR) grading by ophthalmology residents.	Non-Randomized Experimental Study	Ophthalmology; 4 Residents	CNN; Simulations; AI vs. Human performance	The study found no significant difference in five-class DR grading performance with AI (QWK differences: +0.010–0.017, *p* = 0.20–0.74). The PGY-3 resident had improved accuracy (+ 6.0%, *p* = 0.045) and specificity (71.8–80.0%, *p* = 0.019) with AI. AI increased intergrader agreement (FK + 0.072, *p* = 0.003) and confidence in 3 out of 4 residents (*p* < 0.0001).
Pilon [61] 1997; Neural network and linear regression models in residency selection; United States	To compare the effectiveness of linear regression and neural network models in generating provisional rank lists of residency applicants.	Non-Randomized Experimental Study	Emergency Medicine; 74 Residency applicants	Artificial Neural Network; Real world; AI vs. Standard methods	The neural network matched the performance of the linear regression model, achieving correlation coefficients of 0.77 and 0.74, respectively, with R² values of 59.4% for the neural network and 54.0% for the linear regression model.
Quinn [36] 2023; The robot doesn’t lie: real-life validation of robotic performance metrics.; United States	To validate robotic performance metrics against human observation in assessing resident participation during robotic surgeries.	Development & Validation Study	General Surgery; 4 Residents; Faculty	Other: Robotic surgical system (Da Vinci Surgical System); Real world; AI vs. Human performance	Robotic metrics showed a high correlation with human observations (*r* = 0.98, *p* < 0.0001), indicating strong agreement. Residents’ self-assessments and faculty assessments were found to be less accurate.
Reeder [18] 2022; Impact of artificial intelligence on US medical students’ choice of radiology; United States	To examine the impact of AI on US medical students’ choice of radiology as a career, and how such impact is influenced by students’ opinions on and exposures to AI and radiology.	Survey study	Radiology; 463 Medical Students	General AI; Real world; No comparator	AI concerns reduced students’ preference for radiology as a first choice (*P* < 0.001), with 17% deterred by issues like lesser understanding, perceived job reduction, and peer and professional influences. Students preferred AI education during their rotations.
Rees [59] 2023; Machine Learning for The Prediction of Ranked Applicants and Matriculants to an Internal Medicine Residency Program; United States	Evaluate the usefulness of the Random Forest machine learning algorithm to predict ranked applicants and matriculants in an internal medicine residency program	Predictive Study	Medicine; 5067 Residency applicants	Machine Learning; Real world; No comparator	The algorithm was used to predict ranked and matriculated applicants among 5,067 applications to an internal medicine residency program over 3 years. The RF model achieved an AUROC of 0.925 for distinguishing ranked from unranked applicants and 0.597 for identifying matriculants among ranked candidates.
Reich [30] 2022; Artificial Neural Network Approach to Competency-Based Training Using a Virtual Reality Neurosurgical Simulation; Canada	To outline the educational utility of using an ANN in the assessment and quantitation of surgical expertise.	Development & Validation Study	Neurosurgery; Orthopedics; 21 Residents; Fellows; Faculty	Artificial Neural Network; Simulations; No comparator	21 participants were evaluated, and the ANN model, trained on six safety metrics, achieved 83.3% accuracy in classifying expertise levels. ANNs identified continuous and discontinuous learning patterns, showing potential to enhance competency-based surgical training.
Rizzo [82] 2024; The performance of ChatGPT on orthopaedic in-service training exams: A comparative study of the GPT-3.5 turbo and GPT-4 models in orthopaedic education; United States	To investigate the application of LLMs within the realm of orthopaedic in-service training examinations.	Non-Randomized Experimental Study	Orthopedics; No human participant	Large Language Models; Real world; AI vs. other AI tools	GPT-4 consistently outperformed GPT-3.5 Turbo across various years and question categories, achieving 67.63% accuracy in 2022, 58.69% in 2021, and 59.53% in 2020, compared to GPT-3.5 Turbo’s 50.24%, 47.42%, and 46.51% respectively. Both models performed better on non-media questions, with GPT-4 consistently scoring higher on both first-order and higher-order questions.
Ruzicki [38] 2023; Use of Machine Learning to Assess Cataract Surgery Skill Level With Tool Detection; Canada	To develop a method for objective analysis of reproducible steps in routine cataract surgery and distinguish between expert and trainee surgical movements.	Development & Validation Study	Ophthalmology; No human participant	Machine Learning; Other: Deep neural networks; Real world; No comparator	Tool detection achieved high accuracy with AUC ranging from 0.933 to 0.998, while skill classification showed lower accuracy with AUC ranging from 0.550 to 0.692 depending on the scenario.
Ryder [48] 2024; Using Artificial Intelligence to Gauge Competency on a Novel Laparoscopic Training System.; Canada	To develop an NLP model for evaluating the quality of supervisor narrative comments in CBME	Development & Validation Study	Emergency Medicine; 50 Residents; Faculty	Natural Language Processing; Real world; No comparator	The system demonstrated high accuracy in identifying low-quality comments, achieving an 87% accuracy rate within 1 point of the human-rated score.
Salastekar [26] 2023; Artificial Intelligence/Machine Learning Education in Radiology: Multi-institutional Survey of Radiology Residents in the United States.; United States	To evaluate radiology residents perspectives regarding the inclusion of artificial intelligence/machine learning (AI/ML) education in the residency curriculum.	Survey study	Radiology; 209 Residents	Machine Learning; Other: General AI; Real world; No comparator	83% of radiology residents supported integrating AI/ML education into their residency curriculum, preferring hands-on AI/ML laboratories (67%) and lecture series (61%). Most residents favored a continuous AI/ML course spanning the entire residency from R1 to R4.
Sarraf [54] 2021; Use of artificial intelligence for gender bias analysis in letters of recommendation for general surgery residency candidates; United States	Examine gender bias in LoRs written for surgical residency candidates across three decades	Observation Study	General Surgery; 171 Residency Applicants	Natural Language Processing; Other: Sentiment analysis; Real world; No comparator	The study analyzed 611 LoRs using AI to detect gender bias. Gendered wording was more common in LoRs for female applicants (*p* = 0.04), especially those with lower clerkship grades (*p* = 0.01). Sentiment analysis showed male-authored LoRs for male applicants had more positive sentiment (*p* = 0.02). LoRs written before 2000 were shorter but showed no significant gender differences in word count (*p* = 0.18). Gender bias increased over time with more male-biased LoRs.
Sewell [29] 2008; Providing metrics and performance feedback in a surgical simulator.; United States	To present and validate metrics and feedback mechanisms for a mastoidectomy simulator	Observation Study	Otolaryngology; 15 Residents; Faculty; Medical Students	Machine Learning; Simulations; No comparator	HMM’s classified expert and novice surgical performance with 87.5% accuracy. The logistic regression classifier showed high accuracy for metrics like bone visibility (100%) and excessive forces near the facial nerve (87.5%). Feedback mechanisms were validated by significant correlations with instructor-assigned scores, proving their effectiveness in improving surgical skills
Shah [99] 2022; Artificial Intelligence-Powered Clinical Decision Support and Simulation Platform for Radiology Trainee Education.; United States	Investigate the use of AI-based CDS software for automated feedback to radiology trainees	Observation Study	Radiology; 10 Residents; Fellows	Decision support systems; Other: Bayesian inference-based CDS; Simulations; AI vs. Standard methods	Trainees rated the educational value of simulation cases with CDS higher and had slightly lower confidence in their findings compared to clinical cases without CDS (*p* < 0.05). No significant differences were found in timing or ratings of clinical cases with or without CDS
Shiang [9] 2022; Artificial intelligence-based decision support system (AI-DSS) implementation in radiology residency: Introducing residents to AI in the clinical setting.; United States	Evaluate residents’ real-time experiences and perceptions using AI-DSS in the clinical setting and provide recommendations on improving AI curriculums in residency programs.	Survey study	Radiology; 15 Residents	Decision support systems; Other: AI-based decision support system (AI-DSS); Real world; No comparator	91.6% supported AI integration into the curriculum. AI-DSS was found helpful for triaging (83.3%) and troubleshooting (66.7%), but less so for diagnostic purposes (speed: 41.7%, accuracy: 33.3%). Most residents (83.3%) felt positive about AI’s impact on radiology and 50% were motivated to learn more about AI.
Siyar [34] 2018; Using classifiers to distinguish neurosurgical skill levels in a virtual reality tumor resection task; Iran, Canada	To distinguish between skilled and less skilled operators in a virtual reality neurosurgical simulator by applying classifiers.	Observation Study	Neurosurgery; 115 Residents; Fellows; Medical Students	Decision support systems; Simulations; No comparator	The Fuzzy K-Nearest-Neighbors (FKNN) classifier showed the best performance with an average equal error rate (EER) as low as 9.6%.
Smith [84] 2023; Will code one day run a code? Performance of language models on ACEM primary examinations and implications.; Australia	To explore the performance of LLMs on ACEM primary examinations and discuss their implications for medical education and practice.	Non-Randomized Experimental Study	Emergency Medicine; No human participant	Large Language Models; Simulations; AI vs. other AI tools	GPT-4.0 outperformed average candidates, while Bard and Bing achieved passing marks but did not outperform the mean candidate.
Solano [50] 2021; Natural Language Processing and Assessment of Resident Feedback Quality; United States	To validate the performance of a natural language processing (NLP) model in characterizing the quality of feedback provided to surgical trainees.	Development & Validation Study	General Surgery; No human participant	Natural Language Processing; Real world; No comparator	NLP model demonstrated high accuracy (0.83) and specificity (0.97) in classifying feedback quality, with an AUROC of 0.86. However, its sensitivity was relatively low at 0.37.
Spadafore [46] 2024; Using Natural Language Processing to Evaluate the Quality of Supervisor Narrative Comments in Competency-Based Medical Education; Canada	To develop an NLP model to evaluate the quality of supervisor narrative comments in CBME	Development & Validation Study	Emergency Medicine; 50 Residents; Faculty	Machine Learning; Natural Language Processing; Real world; No comparator	QuAL model predicted the exact human-rated score or within one point in 87% of instances. It performed excellently, especially in subtasks on suggestions for improvement and linking resident performance to improvement suggestions, with balanced accuracies of 85% and 82%, respectively.
Stahl [49] 2021; Natural language processing and entrustable professional activity text feedback in surgery: A machine learning model of resident autonomy.; United States	To analyze EPA assessment narrative comments using NLP to enhance understanding of resident entrustment in practice.	Observation Study	General Surgery; Emergency Medicine; 144 Residents; Faculty	Natural Language Processing; Real world; No comparator	Over 18 months, 1,015 EPA microassessments were collected from 64 faculty for 80 residents. LDA analysis identified topics that mapped 1:1 to EPA entrustment levels (Gammas > 0.99), showing coherent trends with high-entrustment words in high-entrustment topics and low-entrustment words in low-entrustment topics.
Summers [60] 2021; Analysis of the Impact of Step 1 Scores on Rank Order for the NRMP Match.; United States	To determine the impact of changing USMLE Step 1 score reporting to Pass/Fail on the rank order of residency applicants.	Predictive Study	Medicine; Not specified Residency applicants	Artificial Neural Network; Real world; No comparator	This study used a deep neural network to model the impact of USMLE Step 1 scores on residency rank order. The model showed a high correlation (0.983) between rank lists with and without Step 1 scores. Key factors affecting ranking were interview scores, evaluation scales, Step 2 scores, and graduation year.
Thanawala [100] 2022; Overcoming Systems Factors in Case Logging with Artificial Intelligence Tools.; United States	To identify and measure the impact of systems and human factors on case logging in general surgery training programs	Observation Study	General Surgery; 171 Residents	Machine Learning; Other: General AI, Reinforcement learning; Real world; AI vs. Human performance	31,385 cases were logged by 171 residents using the platform. Intelligent case logging increased logging rates from 1.44 to 4.77 cases per resident per week (*p* < 0.00001). Even with manual data entry during connectivity pauses, logging increased to 2.85 cases per week (*p* = 0.0002).
Vasan [55] 2023; Letters of recommendations and personal statements for rhinology fellowship: A deep learning linguistic analysis.; United States	To evaluate general and linguistic category differences in Rhinology fellowship letters of recommendation and personal statements between applicant genders and between international medical graduates (IMGs) and US-trained candidates	Observation Study	Rhinology; 56 Fellowship applicants	Natural Language Processing; Other: Deep learning; Real world; No comparator	Female applicants used more words associated with negative emotions, leadership, and feminism. US-trained applicants used more optimistic words, while IMG applicants used more leadership and work-related words.
Vasoya [119] 2019; ReadMI: An Innovative App to Support Training in Motivational Interviewing; United States	To improve the (Motivational Interviewing) MI training process in graduate medical education using a tool (ReadMI) that provides real-time feedback	Development & Validation Study	Medicine; not specified Residents	Natural Language Processing; Other: Deep learning; Real world; AI vs. Standard methods	ReadMI demonstrated high accuracy: 92% in transcript generation, 95% in conversation time reporting, and 92% for both open- and closed-ended questions. It significantly enhanced Motivational Interviewing (MI) training by providing real-time feedback, improving training efficiency and effectiveness.
Webb [69] 2023; Proof of Concept: Using ChatGPT to Teach Emergency Physicians How to Break Bad News.; United States	To demonstrate the potential of ChatGPT in designing realistic clinical scenarios, enabling active roleplay, and delivering effective feedback for teaching physicians how to break bad news	Development & Validation Study	Emergency Medicine; No human participants	Natural Language Processing; Simulations; No comparator	ChatGPT successfully set up realistic training scenarios, enabled roleplay, and provided clear feedback using the SPIKES framework for breaking bad news.
Winkler-Schwartz [33] 2019; Machine Learning Identification of Surgical and Operative Factors Associated with Surgical Expertise in Virtual Reality Simulation; Canada	To identify surgical and operative factors selected by a machine learning algorithm to accurately classify participants by level of expertise in a virtual reality surgical procedure	Observation Study	Neurosurgery; 50 Residents; Fellows; Faculty; Medical Students	Machine Learning; Simulations; No comparator	The K-nearest neighbor algorithm achieved the highest accuracy at 90%. AI identified key metrics related to instrument movement, force, resection accuracy, and bleeding control, highlighting its potential to enhance surgical training and assessment.
Woods [45] 2023; ‘Your comment is not as helpful as it could be.do you still want to submit?’ Using natural language processing to identify the quality of supervisor narrative comments in competency based medical education; Canada	To develop an NLP model for applying the QuAL score to supervisor narrative comments	Development & Validation Study	Emergency Medicine; 50 Residents; Faculty	Natural Language Processing; Real world; No comparator	The NLP model showed reasonable accuracy in rating narrative comments, with balanced accuracies of 0.615 for performance descriptions, 0.85 for suggestions for improvement, and 0.902 for linking performance with suggestions. The overall QuAL score had a balanced accuracy of 0.52 and a top-2 accuracy of 0.83, indicating potential for integration into learning management systems.
Wu [87] 2020; Comparison of Chest Radiograph Interpretations by Artificial Intelligence Algorithm vs. Radiology Residents.; United States	To assess the performance of AI algorithms in realistic radiology workflows by performing an objective comparative evaluation of the preliminary reads of AP frontal chest radiographs by an AI algorithm and radiology residents.	Non-Randomized Experimental Study	Radiology; 5 Residents	Machine Learning; Real world; AI vs. Human performance	The AI achieved a mean image-based sensitivity of 0.716 (95% CI, 0.704–0.729) versus 0.720 (95% CI, 0.709–0.732) for residents (*P* = 0.66). The AI had higher PPV (0.730 vs. 0.682, *P* < 0.001) and specificity (0.980 vs. 0.973, *P* < 0.001). There was no significant difference in sensitivity, but the AI had better specificity and PPV
Wu [20] 2022; Factors Influencing Trainees Interest in Breast Imaging; Canada	To gauge the level of interest in breast imaging (BI) and determine factors impacting traineesâ€™ decision to pursue this subspecialty	Survey study	Radiology; 157 Residents; Medical Students	General AI; Real world; No comparator	36% of residents and 65% of medical students were interested in BI/WI fellowships Trainees disinterested in BI/WI believed that AI will decrease the need for breast radiologists.
Yi [88] 2020; Can AI outperform a junior resident? Comparison of deep neural network to first-year radiology residents for identification of pneumothorax; United States	To develop a deep learning system (DLS) using a deep convolutional neural network (DCNN) for identification of pneumothorax, compare its performance to first-year radiology residents, and evaluate the ability of a DLS to augment radiology residents by detecting missed pneumothoraces.	Development & Validation Study	Radiology; 2 Residents	Other: Deep CNN; Real world; AI vs. Human performance	The DCNN achieved an AUC of 0.841, with a sensitivity of 85% and specificity of 67%, while the residents achieved higher AUCs of 0.942 and 0.905 but at a significantly slower rate (2 images/min vs. 1980 images/min for the DCNN). The DCNN identified 3 additional pneumothoraces missed by the residents
Yi [66] 2023; A novel use of an artificially intelligent Chatbot and a live, synchronous virtual question-and answer session for fellowship recruitment; United States	To determine if an Artificially Intelligent Chatbot and a Virtual Question-and-Answer Session can aid in recruitment in a post-COVID-19 eraâ€‹â€‹.	Survey study	Anesthesiology (Pain fellowship); 48 Fellows	Natural Language Processing; Real world; No comparator	Out of 48 pain fellowship applicants, 18.6% responded. Among respondents, 73% used the website chatbot, and 84% of those reported the chatbot successfully provided the information they were seeking.
Yilmaz [28] 2022; Continuous monitoring of surgical bimanual expertise using deep neural networks in virtual reality simulation; Canada	To develop and validate the Intelligent Continuous Expertise Monitoring System (ICEMS) for assessing surgical bimanual performance in real-time using deep neural networks in a virtual reality simulation.	Development & Validation Study	Neurosurgery; 50 Residents; Fellows; Faculty; Medical Students	Machine Learning; Simulations; No comparator	ICEMS differentiated performance levels among neurosurgeons, senior trainees, junior trainees, and students. Average scores varied significantly (*p* < 0.001), with seniors scoring higher than juniors (mean difference = 0.367, *p* = 0.004) and juniors scoring higher than novices (mean difference = 0.289, *p* = 0.04). Scores correlated with training years, increasing by 0.092 per year (*p* = 0.005).
Yost [44] 2015; Predicting academic performance in surgical training; United States	To determine if residents at risk for substandard performance on the American Board of Surgery In-Training Examination (ABSITE) can be identified based on their behavioral and motivational characteristics.	Predictive Study	General Surgery; 117 Residents	Artificial Neural Network; Real world; AI vs. Human performance	For senior residents, higher theoretical scores were associated with lower pass rates on the ABSITE (*p* = 0.043) while for junior residents, higher internal role awareness scores were associated with higher pass rates (*p* = 0.004). The neural network model accurately predicted ABSITE performance, identifying key behavioral and motivational factors.
Zhang [47] 2012; Automated assessment of medical training evaluation text.; United States	To assess the feasibility and value of an automated approach for synthesizing evaluation comments of residency trainees using text-mining techniques for sentiment and topic analysis	Development & Validation Study	Medicine; Pediatrics; No human participant	Machine Learning; Natural Language Processing; Real world; No comparator	SVM achieved 93% accuracy in sentiment analysis. For topic classification, performance varied by competency, with an overall precision and recall of 76.3%.
Zhao [67] 2020; Comparison of Multiple Quantitative Evaluation Indices of Theoretical Knowledge and Clinical Practice Skills and Training of Medical Interns in Cardiovascular Imaging Using Blended Teaching and the Case Resource Network Platform (CRNP).; China	To compare multiple quantitative evaluation indices of theoretical knowledge and clinical practice skills in training medical interns in cardiovascular imaging using blended teaching (BT) and the Case Resource Network Platform (CRNP)	Observation Study	Cardiology; 110 Residents	Other: Case Resource Network Platform (CRNP) integrating artificial intelligence; Real world; AI vs. Standard methods	The BT group showed significant improvements in CT angiography scores (6.53 vs. 5.76, *p* = 0.022), cardiac MRI scores (5.69 vs. 4.73, *p* = 0.016), and average scores (6.32 vs. 5.69, *p* = 0.002).
Zhao [89] 2020; Reducing the number of unnecessary biopsies of US-BI-RADS 4a lesions through a deep learning method for residents-in-training: a cross-sectional study.; China	To explore the potential value of S-Detect for residents-in-training, a computer-assisted diagnosis system based on a deep learning algorithm	Observation Study	Radiology; 195 focal breast lesions (patients) Residents	Deep learning; Real world; AI vs. Human performance	S-Detect tool demonstrated high specificity (77.88%) and sensitivity (85.37%), with an AUC of 0.82, indicating better specificity compared to residents who had high sensitivity but lower specificity

Abbreviations used: ABSITE: American Board of Surgery In-Training Examination, AI: Artificial Intelligence, ANN: Artificial Neural Network, AUC: Area Under the Curve, CCC: Clinical Competency Committee, CNN: Convolutional Neural Network, CT: Computed Tomography, DLS: Deep Learning System, DNN: Deep Neural Network, EMR: Electronic Medical Record, EPA: Entrustable Professional Activity, GME: Graduate Medical Education, GOALS: Global Operative Assessment of Laparoscopic Skills, ITE: In-Training Examination, JBI: Joanna Briggs Institute, LLM: Large Language Model, LOR: Letter of Recommendation, MI: Motivational Interviewing, ML: Machine Learning, NER: Named Entity Recognition, NLI: Natural Language Interpretation, NLP: Natural Language Processing, NRMP: National Resident Matching Program, OBGYN: Obstetrics and Gynecology, OITE: Orthopaedic In-Training Examination, PRISMA: Preferred Reporting Items for Systematic Reviews and Meta-Analyses, RNN: Recurrent Neural Network, SVM: Support Vector Machine, SVR: Support Vector Regression, US: Ultrasound, USMLE: United States Medical Licensing Examination, VR: Virtual Reality

The most common study types were development and validation studies [32], observational studies [26], non-randomized experimental studies [16], survey studies [14], and predictive studies [10]. Only one randomized controlled trial was identified and one development study without validation. These studies explored a wide range of AI applications, from skill assessment tools and diagnostic support to recruitment analytics and educational interventions. Further details on their specific findings and implications are discussed below.

### Application of AI in GME

#### Adoption, perception, and attitudes towards AI

Out of twelve studies reviewed, nine focused on radiology. Concerns about AI influencing specialty choices were evident, with one survey reporting a significant decrease in radiology’s appeal as a first-choice specialty among medical students (*P* < 0.001), deterring 17% of respondents [18]. Concerns about job security due to AI were also noted [19]. Similarly, radiology and oncology residents expressed worries about AI reducing the demand for professionals in their fields [20, 21]. Huisman [22] found that limited knowledge about AI often leads to fear of job replacement, whereas intermediate to advanced knowledge fosters positive attitudes. Younger individuals, those with scientific backgrounds, and active social media users tended to view AI more favorably.

Recent studies [23, 24] indicate a shift in perception among radiology residents, with most now viewing AI positively. According to one study, 76% of residents find AI exciting, 80% would still choose radiology as a specialty, and 74% see no threat to job security from AI [23]. Marquis [25] summarized a 2020 survey by the American Alliance of Academic Chief Residents in Radiology, reporting that 74% of respondents believe AI poses no threat to the job market and could enhance workflow efficiency. However, despite 95% of residents recognizing the importance of AI and machine learning education, 20% of training programs still lack formal AI education, as highlighted in a study by Salastekar [26].

There was one study on faculty perception wherein anesthesiology faculty broadly agree that AI will benefit patient care (80%), enhance resident training (86.7%), and improve post-graduate medical education (81.4%), but 67% were concerned about accountability for AI inaccuracies [27].

#### Role of AI in formative & summative assessment

Research has increasingly focused on AI’s impact on both formative and summative assessments in medical education. For instance, the Intelligent Continuous Expertise Monitoring System (ICEMS) successfully identified different levels of surgical performance among neurosurgeons, trainees, and students in a virtual reality (VR) simulation, with performance levels strongly correlating to years of training (*p* = 0.005) [28]. This was also noted similarly in other surgical specialties [29–35] highlighting the efficacy of AI in differentiating skill levels in surgical training, indicating AI’s potential to improve the accuracy of resident training evaluations. In robotic surgery, Quinn [36] validated robotic performance metrics against human observation, showing a high correlation (*r* = 0.98, *p* < 0.0001),

AI’s potential for real-time assessment is also significant. Anh [37] compared feature extraction techniques for automated surgical skill assessment using motion analysis, showing promising results. Likewise, Ruzicki [38] reported high tool detection accuracy (AUC 0.933 to 0.998) in cataract surgery, although skill classification accuracy varied. Similar results were also noted in studies by Holden [39] & Oropesa [40] while Kumar [41] developed an objective framework for robotic surgery training, offering valuable feedback.

AI has also been used for predictions, with Ariaeinejad [42] using a machine learning algorithm to identify performance patterns, achieving a sensitivity of 0.54, specificity of 0.74, and AUC of 0.64. Additonally, studies by Amirhajlou [43] and Yost [44] using neural networks to predict board certification exam performance based on ITE and ABSITE scores, achieving high accuracy.

#### AI in trainee & faculty evaluations

Woods [45] and Spadafore [46] developed NLP models to assess supervisor narrative comments in competency-based medical education. Woods’ model showed balanced accuracies of 0.615 for performance descriptions, 0.85 for suggestions for improvement, and 0.902 for linking performance. Similarly, Spadafore’s model predicted the exact human-rated score or within one point in 87% of instances, with balanced accuracies of 85% for suggestions for improvement and 82% for linking performance. Zhang [47] extended these efforts by assessing text-mining techniques to synthesize evaluation comments for residents, achieving 93% accuracy in sentiment analysis and 76.3% precision and recall for topic classification. Similarly, Ryder [48] developed an NLP system to evaluate supervisor narrative comments, achieving 87% accuracy within 1 point of the human-rated score.

Several studies have focused on enhancing feedback analysis using NLP. Stahl [49] used NLP to analyze EPA assessment narrative comments, enhancing understanding of resident entrustment in practice. Solano [50] validated an NLP model for characterizing feedback quality to surgical trainees, demonstrating high accuracy (83%) and specificity (97%), though with low sensitivity (37%). In a similar vein, Neves [51] employed machine learning to evaluate feedback on anesthesiology resident performance, achieving 74.4-82.2% accuracy in predicting feedback traits and high precision in low-quality predictions while processing data significantly faster than manual scoring.

Other studies have leveraged NLP for broader educational outcomes. For example, Lui [52] used NLP to track medical residents’ cognitive maturation, correlating linguistic markers with faculty assessments, providing insights into their developmental progress.

#### AI in GME recruitment

AI has been used to analyze Letters of Recommendation (LORs), applications, personal statements, as well as to predict rankings or interview invitations and recruitment outcomes.

In studies on LORs, Boolchandani [53] found no significant differences in language based on gender or race. However, Sarraf [54] observed differences in how language was used to describe male and female general surgery candidates, indicating gender-related variations in linguistic style. Additionally, Vasan [55] identified distinct language patterns between U.S.-trained applicants and international medical graduates (IMGs) in the field of rhinology. Gray [56] noted bias in sentiment, with Black applicants receiving the highest positive sentiment and Hispanic applicants the lowest (*p* = 0.03).

In residency applications analysis, Drum [57] showed ML’s moderate sensitivity (0.64) and high specificity (0.97) in identifying values from unstructured data, correlating with interview invitations and enhancing equity. Burk-Rafel’s [58] ML-based decision support tool identified 20 overlooked candidates through holistic screening. AI also predicted applicant rankings [59–62] and interview invitations [63].

Johnstone [64] found that AI-generated personal statements were challenging to distinguish from human-written ones, with 70% of program directors unable to detect a difference. Patel [65] reported no significant difference in ratings for readability, originality, authenticity, and overall quality (all *P* > 0.05) between computer-generated and applicant essays. However, raters were less inclined to grant interviews to computer-generated essays (58% vs. 78%, *P* = 0.12).

AI’s role extends beyond document analysis to interactive tools aimed at enhancing the recruitment experience. For example, Yi [66] highlighted AI’s potential for recruitment through a chatbot for virtual Q&A, suggesting it improved program perception.

#### AI in medical education and training

Bond [10] found that incorporating AI in virtual standardized patient (VSP) simulations significantly increased clinical training effectiveness, with learners accurately diagnosing 82% of cases and boosting their decision-making confidence. Similarly, Zhao [67] reported that a blended teaching approach improved overall learning engagement while Merritt [68] further suggested that AI technology could overcome common obstacles of conventional simulation methods. For example, Webb [69] demonstrated ChatGPT’s potential in designing realistic clinical scenarios for training emergency physicians in breaking bad news.

El Saadawi [70] showed AI-based tutoring systems deliver customized educational experiences by accurately interpreting learner inputs. Kelahan [71], supported by Lin [72] and Muntean [73], demonstrated AI’s ability to adapt to learners’ progress, providing personalized feedback and enhancing learner experience. Chen [74] described an NLP-incorporated dashboard to track resident caseloads, identifying training gaps and improving education.

#### AI in standardized examinations

Several studies tested the performance of AI tools in standardized multiple-choice examinations across various medical subspecialties.

Nori [75] investigated GPT-4’s capabilities on medical challenge problems, surpassing the USMLE passing score by over 20 points. Ali [76] assessed GPT-3.5, GPT-4, and Google Bard on a neurosurgery oral boards preparation question bank, with GPT-4 achieving the highest accuracy at 82.6%. Bartoli [77] evaluated ChatGPT in generating and answering neurosurgical written exam questions, ranking 6th out of 11 participants. Gupta [78] applied GPT-4 to the Plastic Surgery Inservice Training Examination, achieving 77.3% accuracy, while Humar [79] found ChatGPT performed on par with a first-year resident on the Plastic Surgery In-Service Examination, but lower than more advanced residents. Holmes [80] compared LLM performance in ophthalmology questions, with GPT-4 performing comparably to attending physicians. Cohen [81] evaluated ChatGPT’s performance in Hebrew OBGYN exams, scoring 38.7% compared to 68.4% by residents, and found it performed better on English medical tests (60.7% accuracy).

Rizzo [82] compared GPT-3.5 Turbo and GPT-4 on orthopaedic in-service training exams, with GPT-4 consistently outperforming GPT-3.5 Turbo while Lum [83] found it performed below passing thresholds for higher years of training. Smith [84] explored LLM performance on ACEM primary examinations, finding GPT-4 outperformed average candidates. Mahajan [85] assessed ChatGPT’s performance on the Otolaryngology Residency In-Service Exam, with accuracy varying by question difficulty.

AI has also been studied to generate questions relevant for GME. Cheung [86] found AI-generated MCQs matched human-created ones in quality across most domains but were slightly inferior in relevance. ChatGPT notably produced MCQs much faster than humans.

#### AI in clinical decision making and training

Wu [87] and Yi [88] compared the diagnostic abilities of AI and resident physicians. Wu found that AI’s performance on chest radiographs was comparable to that of residents while, Yi noted that a deep learning system analyzed pneumothorax images about 1000 times faster, potentially reducing misdiagnosis and enhancing training.

AI has also been used in different specialties and scenario to improve diagnostic accuracy and confidence of residents and fellows [89–96]. For example, Lee [97] used a deep learning model in the CT diagnosis of cervical lymph node metastasis in thyroid cancer to help underperforming residents. Chassagnon [98] reported that a computer-aided detection (CADe) system improved the diagnostic sensitivity, specificity, and accuracy of residents with active use of the system. Interestingly, Shah [99] found that simulation cases with Clinical Decision Support (CDS) were valued educationally, but resulted in lower confidence in findings and diagnosis. Shiang [9] highlighted that radiology residents supported AI-DSS for triaging patients and troubleshooting tasks.

AI use has also been studied in other aspects of GME workflow. For example, Thanawala [100] showed AI increased case logging efficiency in general surgery training programs, while Gong explored AI’s potential in analyzing clinical notes to reduce work hours. Gao [102] and Ouyang [103] leveraged historical medical records to enhance diagnostic learning for interns and novice physicians.

## Limitations identified in various studies

The reviewed studies reported several limitations that affected the generalizability and accuracy of their findings. Key methodological concerns include self-reporting biases, low response rates, and survey design challenges [18, 19, 22, 23, 68, 104]. Additionally, small sample sizes were a recurrent issue. For example, Bond et al. [10] tested AI-driven virtual standardized patient simulations on only 14 residents, limiting the ability to draw broad conclusions about its effectiveness across different specialties. Similarly, studies assessing AI’s predictive ability for board certification exams [43, 44] often used data from a single institution or a limited cohort, making it difficult to apply findings universally.

Another critical limitation is the variability in AI applications, which affects comparability across studies. AI tools used for predicting board exam performance [42, 43] rely on structured data inputs, whereas AI applications in clinical decision-making [86, 87, 97] involve deep learning models trained on imaging datasets. This fundamental difference in AI methodologies means findings from one domain cannot be extrapolated to another. For instance, an AI system that performs well in surgical skill assessment [28, 29] using motion tracking may not translate to radiology decision support systems [88, 89].

Furthermore, algorithmic biases are a recurring issue. Sarraf et al. [54] identified gender-related variations in how AI analyzed letters of recommendation for surgery applicants, raising concerns about fairness in recruitment. Similarly, studies applying NLP to performance evaluations [45, 46] identified potential bias in feedback interpretation, which could reinforce pre-existing disparities in medical training.

The ongoing refinement and validation of AI technologies are frequently recommended because of performance concerns [56, 58, 59, 64, 92, 102, 105, 106]. Many AI models are developed in controlled environments but require further testing in real-world clinical settings to ensure robustness.

Clinical context limitations and participant-related issues highlight the challenges in applying AI across diverse clinical settings and populations [33, 40, 41, 81, 86, 107]. For example, an AI-driven competency assessment tool tested in a single specialty or training site may not be effective when applied to a multispecialty residency program with different educational needs.

## Implications for future research and practice

Future research should validate current findings and explore additional AI applications for GME. Studies need large sample sizes, multiple sites, and comprehensive data to enhance generalizability and reliability. Evaluating and improving AI models, including large language models (LLMs) and neural networks, is crucial. Addressing algorithmic biases identified in the reviewed studies is essential for ensuring fairness and accuracy.

While this review summarizes predictive accuracy metrics (e.g., AUC, sensitivity, specificity), direct comparisons to established clinical benchmarks are beyond the scope of a scoping review. Future studies should systematically assess the clinical significance of AI models by comparing them against human performance standards or widely accepted diagnostic thresholds.

In addition to addressing biases, ensuring fair representation, including gender and nonbinary considerations, is essential. Algorithmic biases in recruitment, assessment, and performance evaluation may perpetuate disparities, making fairness audits and model validation crucial before widespread implementation. Ethical considerations such as data privacy, informed consent, and transparency in AI algorithms must be prioritized to maintain the effectiveness and reliability of AI-driven tools. AI systems trained on clinical or educational data pose risks of data misuse, necessitating strong regulatory oversight to ensure compliance with privacy laws (e.g., HIPAA, GDPR). Additionally, questions of accountability remain unresolved—when AI contributes to residency evaluations or patient care decisions, clear guidelines are needed to delineate responsibility between AI developers, educators, and clinicians.

Given the gap in AI training within residency programs, incorporating AI education into curricula is recommended along with faculty training for effectively utilizing AI tools and interpreting AI-generated data. One approach could be adapting the competency-based medical education (CBME) framework, which is widely used in medical education, to include AI skills alongside clinical competencies. For instance, Tippur [108] discusses how AI education can be integrated into curricula, focusing on bridging the gap between current training methodologies and the evolving technological landscape. Similarly, other fields have successfully integrated AI education into their curricula. Southworth [109] presented a model for embedding AI across the higher education curriculum, highlighting the importance of AI literacy for future professionals, which could serve as a valuable framework for GME programs.

Research should also explore AI applications in broader aspects of medical education. This includes tailoring teaching to individual learning needs, optimizing educational outcomes and providing personalized feedback. Longitudinal studies are needed to assess the long-term impact of AI tools on clinical competencies and patient outcomes to understand AI’s broader implications in GME.

AI’s role in resident and fellow recruitment and selection processes shows promise but also highlights biases and disparities. Future studies should refine AI algorithms to ensure fairness and equity in recruitment. Exploring AI integration in holistic review processes can further enhance fairness.

Finally, developing standardized evaluation metrics to assess AI interventions will ensure consistency across studies. Evaluating the cost-effectiveness of AI applications, considering both initial investment and long-term benefits, is essential. Future research should explore how AI can support trainees in preparing for standardized exams and improving their test performance.

## Limitations of this scoping review

Study heterogeneity in design, AI applications, and outcome measures posed a significant challenge to synthesis. The included studies varied in methodology, participant populations, and AI implementation strategies, making direct comparisons difficult. Some studies focused on predictive modeling, while others assessed AI’s role in education, evaluation, or clinical decision-making, leading to variability in reported effectiveness and applicability. This heterogeneity necessitated a descriptive rather than a comparative synthesis, limiting our ability to draw definitive conclusions about AI’s overall impact on GME.

To mitigate this challenge in future research, efforts should be made to standardize study designs and reporting frameworks. Establishing common evaluation metrics for AI interventions in GME would improve comparability across studies. Additionally, multi-institutional collaborations using shared methodologies can enhance the generalizability of findings. Implementing structured reporting guidelines—such as those used in AI applications in clinical medicine—could further reduce inconsistencies in study outcomes.

While this review provides a broad overview of AI’s applications in GME, the rapidly evolving nature of AI necessitates ongoing updates to maintain relevance. Future research should not only focus on validating findings across multiple settings but also on developing standardized methodologies that facilitate clearer synthesis of AI’s role in medical education.

## Conclusion

This scoping review provides a comprehensive overview of the applications, benefits, and challenges of AI integration in GME. However, study heterogeneity, small sample sizes, and limited multi-institutional validation hinder generalizability. Additionally, biases in AI models, particularly in recruitment and performance evaluation, highlight the need for fairness audits and robust validation before widespread implementation.

Future research should focus on standardized evaluation frameworks for AI-driven assessments, ensuring their reliability and applicability across diverse training environments. Addressing the gap in AI literacy among trainees and faculty is also critical, with competency-based educational models offering a potential pathway for structured AI integration into curricula. Additionally, refining AI algorithms to promote equity in residency recruitment and performance assessments remains a crucial area for ongoing investigation.

As AI continues to evolve, periodic re-evaluations of its impact on GME will be necessary to ensure its benefits outweigh risks. Expanding research efforts across multiple specialties and global settings will provide more inclusive insights, ultimately guiding the responsible adoption of AI in GME.

## Data Availability

The datasets used and/or analyzed during the current study are available from the corresponding author on reasonable request.

## References

[1] LeCun Y, Bengio Y, Hinton G. Deep learning. Nature. 2015;521(7553):436–44. 26017442 10.1038/nature14539

[2] Murphy R. Introduction to AI robotics. Cambridge, MA: The MIT Press; 2018.

[3] Amisha, Malik P, Pathania M, Rathaur VK. Overview of artificial intelligence in medicine. J Fam Med Prim Care. 2019;8(7):2328–31. 10.4103/jfmpc.jfmpc_440_19 PMC6691444 31463251

[4] Van Der Niet AG, Bleakley A. Where medical education Meets artificial intelligence: ‘does technology care?’. Med Educ. 2021;55(1):30–6. 32078175 10.1111/medu.14131

[5] Nagi F, Salih R, Alzubaidi M, Shah H, Alam T, Shah Z, et al. Applications of artificial intelligence (AI) in medical education: A scoping review. Stud Health Technol Inf. 2023;305:648–51. 10.3233/SHTI230581 37387115

[6] Maldonado ME, Fried ED, DuBose TD, Nelson C, Breida M. The role that graduate medical education must play in ensuring health equity and eliminating health care disparities. Ann Am Thorac Soc. 2014;11(4):603–7. 24708150 10.1513/AnnalsATS.201402-068PS

[7] Stawicki P, Kumar S, Firstenberg KNS, Orlando MP, Papadimos JJ, Paul T et al. E, Introductory Chapter: Navigating Challenges and Opportunities in Modern Graduate Medical Education. In: P. Stawicki S, S. Firstenberg M, P. Orlando J, J. Papadimos T, editors. Contemporary Topics in Graduate Medical Education - Volume 2. IntechOpen; 2022 [cited 2024 Jan 8]. Available from: https://www.intechopen.com/chapters/79744

[8] Boms O, Shi Z, Mallipeddi N, Chung JJ, Marks WH, Whitehead DC, et al. Integrating innovation as a core objective in medical training. Nat Biotechnol. 2022;40(3):434–7. 35296823 10.1038/s41587-022-01253-x

[9] Shiang T, Garwood E, Debenedectis CM. Artificial intelligence-based decision support system (AI-DSS) implementation in radiology residency: introducing residents to AI in the clinical setting. Clin Imaging. 2022;92:32–7. 36183619 10.1016/j.clinimag.2022.09.003

[10] Bond WF, Lynch TJ, Mischler MJ, Fish JL, McGarvey JS, Taylor JT, et al. Virtual standardized patient simulation: case development and pilot application to High-Value care. Simul Healthc J Soc Simul Healthc. 2019;14(4):241–50. 10.1097/SIH.0000000000000373 31116172

[11] Lee J, Wu AS, Li D, Kulasegaram K (Mahan), editors. Artificial Intelligence in Undergraduate Medical Education: A Scoping Review. Acad Med. 2021;96(11S):S62–70. 10.1097/ACM.0000000000004291 34348374

[12] Kirubarajan A, Young D, Khan S, Crasto N, Sobel M, Sussman D. Artificial intelligence and surgical education: A systematic scoping review of interventions. J Surg Educ. 2022;79(2):500–15. 34756807 10.1016/j.jsurg.2021.09.012

[13] Abdel Aziz MH, Rowe C, Southwood R, Nogid A, Berman S, Gustafson K. A scoping review of artificial intelligence within pharmacy education. Am J Pharm Educ. 2023;100615. 10.1016/j.ajpe.2023.100615 37914030

[14] Arksey H, O’Malley L. Scoping studies: towards a methodological framework. Int J Soc Res Methodol. 2005;8(1):19–32.

[15] Peters MDJ, Godfrey CM, Khalil H, McInerney P, Parker D, Soares CB. Guidance for conducting systematic scoping reviews. Int J Evid Based Healthc. 2015;13(3):141–6. 26134548 10.1097/XEB.0000000000000050

[16] Tricco AC, Lillie E, Zarin W, O’Brien KK, Colquhoun H, Levac D, et al. PRISMA extension for scoping reviews (PRISMA-ScR): checklist and explanation. Ann Intern Med. 2018;169(7):467–73. 30178033 10.7326/M18-0850

[17] Sheehy R, White J, Verghese B, Iyer C. Protocol for A Scoping Review of Artificial Intelligence in Graduate Medical Education. OSF Registries; 2024 [cited 2024 Jul 16]. Available from: https://osf.io/uw2n7/

[18] Reeder K, Lee H. Impact of artificial intelligence on US medical students’ choice of radiology. Clin Imaging. 2022;81:67–71. 34619566 10.1016/j.clinimag.2021.09.018

[19] Collado-Mesa F, Alvarez E, Arheart K. The role of artificial intelligence in diagnostic radiology: A survey at a single radiology residency training program. J Am Coll Radiol. 2018;15(12):1753–7. 29477289 10.1016/j.jacr.2017.12.021

[20] Wu T, Law W, Islam N, Yong-Hing CJ, Kulkarni S, Seely J. Factors influencing trainees’ interest in breast imaging. Can Assoc Radiol J. 2022;73(3):462–72. 34913752 10.1177/08465371211049553

[21] Kennedy T, Collie L, Nabhen J, Safavi A, Brundage M, De Moraes FY. 136: Canadian oncology residents’ knowledge of and attitudes towards artificial intelligence and machine learning. Radiother Oncol. 2022;174:S58–9.

[22] Huisman M, Ranschaert E, Parker W, Mastrodicasa D, Koci M, Pinto De Santos D, et al. An international survey on AI in radiology in 1,041 radiologists and radiology residents part 1: fear of replacement, knowledge, and attitude. Eur Radiol. 2021;31(9):7058–66. 33744991 10.1007/s00330-021-07781-5 PMC8379099

[23] Chen Y, Wu Z, Wang P, Xie L, Yan M, Jiang M, et al. Radiology residents’ perceptions of artificial intelligence: nationwide Cross-Sectional survey study. J Med Internet Res. 2023;25:e48249. 37856181 10.2196/48249 PMC10623237

[24] Ooi S, Makmur A, Soon Y, Fook-Chong S, Liew C, Sia D, et al. Attitudes toward artificial intelligence in radiology with learner needs assessment within radiology residency programmes: a National multi-programme survey. Singap Med J. 2021;62(3):126–34. 10.11622/smedj.2019141 PMC8027147 31680181

[25] Marquis KM, Hoegger MJ, Shetty AS, Bishop GL, Balthazar P, Gould JE, et al. Results of the 2020 survey of the American alliance of academic chief residents in radiology. Clin Imaging. 2023;98:67–73. 37023549 10.1016/j.clinimag.2023.02.008

[26] Salastekar NV, Maxfield C, Hanna TN, Krupinski EA, Heitkamp D, Grimm LJ. Artificial intelligence/machine learning education in radiology: Multi-institutional survey of radiology residents in the united States. Acad Radiol. 2023;30(7):1481–7. 36710101 10.1016/j.acra.2023.01.005

[27] Kocer Tulgar Y, Department of Medical History and Ethics, Medicine SU, Turkey S, Tulgar S, Department of Anaesthesiology and Reanimation, Samsun University Faculty of Medicine, Samsun Training and Research Hospital, Samsun, Turkey, Kose G, Kose S et al. HC,. Anesthesiologists’ Perspective on the Use of Artificial Intelligence in Ultrasound-Guided Regional Anaesthesia in Terms of Medical Ethics and Medical Education: A Survey Study. Eurasian J Med. 2023 May 5 [cited 2024 May 18]; Available from: https://www.eajm.org//en/anesthesiologists-perspective-on-the-use-of-artificial-intelligence-in-ultrasound-guided-regional-anaesthesia-in-terms-of-medical-ethics-and-medical-education-a-survey-study-133475 10.5152/eurasianjmed.2023.22254 PMC10440966 37161553

[28] Yilmaz R, Winkler-Schwartz A, Mirchi N, Reich A, Christie S, Tran DH, et al. Continuous monitoring of surgical bimanual expertise using deep neural networks in virtual reality simulation. Npj Digit Med. 2022;5(1):54. 35473961 10.1038/s41746-022-00596-8 PMC9042967

[29] Sewell C, Morris D, Blevins NH, Dutta S, Agrawal S, Barbagli F, et al. Providing metrics and performance feedback in a surgical simulator. Comput Aided Surg. 2008;13(2):63–81. 18317956 10.3109/10929080801957712

[30] Reich A, Mirchi N, Yilmaz R, Ledwos N, Bissonnette V, Tran DH, et al. Artificial neural network approach to Competency-Based training using a virtual reality neurosurgical simulation. Oper Neurosurg. 2022;23(1):31–9. 10.1227/ons.0000000000000173 35726927

[31] Alkadri S, Ledwos N, Mirchi N, Reich A, Yilmaz R, Driscoll M, et al. Utilizing a multilayer perceptron artificial neural network to assess a virtual reality surgical procedure. Comput Biol Med. 2021;136:104770. 34426170 10.1016/j.compbiomed.2021.104770

[32] Baloul MS, Yeh VJH, Mukhtar F, Ramachandran D, Traynor MD, Shaikh N, et al. Video commentary & machine learning: tell me what you see, I tell you who you are. J Surg Educ. 2022;79(6):e263–72. 33077418 10.1016/j.jsurg.2020.09.022

[33] Winkler-Schwartz A, Yilmaz R, Mirchi N, Bissonnette V, Ledwos N, Siyar S, et al. Machine learning identification of surgical and operative factors associated with surgical expertise in virtual reality simulation. JAMA Netw Open. 2019;2(8):e198363. 31373651 10.1001/jamanetworkopen.2019.8363

[34] Siyar S, Azarnoush H, Rashidi S, Winkler-Schwartz A. Using classifiers to distinguish neurosurgical skill levels in a virtual reality tumor resection task. Int J Comput Assist Radiol Surg. 2018;13(S1):1–273.29766372

[35] Bissonnette V, Mirchi N, Ledwos N, Alsidieri G, Winkler-Schwartz A, Del Maestro RF, et al. Artificial intelligence distinguishes surgical training levels in a virtual reality spinal task. J Bone Jt Surg. 2019;101(23):e127. 10.2106/JBJS.18.01197 PMC7406145 31800431

[36] Quinn KM, Chen X, Runge LT, Pieper H, Renton D, Meara M, et al. The robot doesn’t Lie: real-life validation of robotic performance metrics. Surg Endosc. 2023;37(7):5547–52. 36266482 10.1007/s00464-022-09707-8

[37] Anh NX, Nataraja RM, Chauhan S. Towards near real-time assessment of surgical skills: A comparison of feature extraction techniques. Comput Methods Programs Biomed. 2020;187:105234. 31794913 10.1016/j.cmpb.2019.105234

[38] Ruzicki J, Holden M, Cheon S, Ungi T, Egan R, Law C. Use of machine learning to assess cataract surgery skill level with tool detection. Ophthalmol Sci. 2023;3(1):100235. 36444216 10.1016/j.xops.2022.100235 PMC9700302

[39] Holden MS, Xia S, Lia H, Keri Z, Bell C, Patterson L, et al. Machine learning methods for automated technical skills assessment with instructional feedback in ultrasound-guided interventions. Int J Comput Assist Radiol Surg. 2019;14(11):1993–2003. 31006107 10.1007/s11548-019-01977-3

[40] Oropesa I, Sánchez-González P, Chmarra MK, Lamata P, Pérez-Rodríguez R, Jansen FW, et al. Supervised classification of psychomotor competence in minimally invasive surgery based on instruments motion analysis. Surg Endosc. 2014;28(2):657–70. 24122243 10.1007/s00464-013-3226-7

[41] Kumar R, Jog A, Vagvolgyi B, Nguyen H, Hager G, Chen CCG, et al. Objective measures for longitudinal assessment of robotic surgery training. J Thorac Cardiovasc Surg. 2012;143(3):528–34. 22172215 10.1016/j.jtcvs.2011.11.002 PMC3288290

[42] Ariaeinejad A, Samavi DR. A Performance Predictive Model for Emergency Medicine Residents.

[43] Amirhajlou L, Sohrabi Z, Alebouyeh MR, Tavakoli N, Haghighi RZ, Hashemi A et al. Application of data mining techniques for predicting residents’ performance on pre–board examinations: A case study. J Educ Health Promot. 2019;8. 10.4103/jehp.jehp_394_18 PMC6615122 31334260

[44] Yost MJ, Gardner J, Bell RM, Fann SA, Lisk JR, Cheadle WG, et al. Predicting academic performance in surgical training. J Surg Educ. 2015;72(3):491–9. 25600356 10.1016/j.jsurg.2014.11.013

[45] Woods R, Spadafore M, Yilmaz Y, Rally V, Russell M, Thoma B, et al. Your comment is not as helpful as it could be… do you still want to submit?’ using natural Language processing to identify the quality of supervisor narrative comments in competency based medical education. Can J Emerg Med. 2023;25(S1):S47. 10.1097/ACM.0000000000005634 38232079

[46] Spadafore M, Yilmaz Y, Rally V, Chan TM, Russell M, Thoma B, et al. Using natural Language processing to evaluate the quality of supervisor narrative comments in Competency-Based medical education. Acad Med. 2024;99(5):534–40. 38232079 10.1097/ACM.0000000000005634

[47] Zhang R. Automated Assessment of Medical Training Evaluation Text. PMC3540577 23304426

[48] Ryder CY, Mott NM, Gross CL, Anidi C, Shigut L, Bidwell SS, et al. Using artificial intelligence to gauge competency on a novel laparoscopic training system. J Surg Educ. 2024;81(2):267–74. 38160118 10.1016/j.jsurg.2023.10.007

[49] Stahl CC, Jung SA, Rosser AA, Kraut AS, Schnapp BH, Westergaard M, et al. Natural Language processing and entrustable professional activity text feedback in surgery: A machine learning model of resident autonomy. Am J Surg. 2021;221(2):369–75. 33256944 10.1016/j.amjsurg.2020.11.044 PMC7969407

[50] Solano QP, Hayward L, Chopra Z, Quanstrom K, Kendrick D, Abbott KL, et al. Natural Language processing and assessment of resident feedback quality. J Surg Educ. 2021;78(6):e72–7. 34167908 10.1016/j.jsurg.2021.05.012

[51] Neves SE, Chen MJ, Ku CM, Karan S, DiLorenzo AN, Schell RM, et al. Using machine learning to evaluate attending feedback on resident performance. Anesth Analg. 2021;132(2):545–55. 33323789 10.1213/ANE.0000000000005265

[52] Lui A, Chary M, Yoneda N, Parikh S. Tracking resident cognitive maturation with natural language processing. West J Emerg Med., (Lui A, Chary M, Yoneda N, Parikh S.) New York Presbyterian Queens, Flushing, NY, United States):S46.

[53] Boolchandani H, Osborn R, Tiyyagura G, Sheares B, Chen L, Phatak UP, et al. Words used in letters of recommendation for pediatric residency applicants: demographic differences and impact on interviews. Acad Pediatr. 2023;23(8):1614–9. 36889506 10.1016/j.acap.2023.02.012

[54] Sarraf D, Vasiliu V, Imberman B, Lindeman B. Use of artificial intelligence for gender bias analysis in letters of recommendation for general surgery residency candidates. Am J Surg. 2021;222(6):1051–9. 34674847 10.1016/j.amjsurg.2021.09.034

[55] Vasan V, Cheng C, Lerner DK, Signore AD, Schaberg M, Govindaraj S, et al. Letters of recommendations and personal statements for rhinology fellowship: A deep learning linguistic analysis. Int Forum Allergy Rhinol. 2023;13(10):1971–3. 36896816 10.1002/alr.23153

[56] Gray GM, Williams SA, Bludevich B, Irby I, Chang H, Danielson PD, et al. Examining implicit Bias differences in pediatric surgical fellowship letters of recommendation using natural Language processing. J Surg Educ. 2023;80(4):547–55. 36529662 10.1016/j.jsurg.2022.12.002

[57] Drum B, Shi J, Peterson B, Lamb S, Hurdle JF, Gradick C. Using natural Language processing and machine learning to identify internal Medicine–Pediatrics residency values in applications. Acad Med. 2023;98(11):1278–82. 37506388 10.1097/ACM.0000000000005352

[58] Burk-Rafel J, Reinstein I, Feng J, Kim MB, Miller LH, Cocks PM, et al. Development and validation of a machine Learning-Based decision support tool for residency applicant screening and review. Acad Med. 2021;96(11S):S54–61. 34348383 10.1097/ACM.0000000000004317

[59] Rees CA, Ryder HF. Machine learning for the prediction of ranked applicants and matriculants to an internal medicine residency program. Teach Learn Med. 2023;35(3):277–86. 35591808 10.1080/10401334.2022.2059664

[60] Summers JA. Analysis of the impact of step 1 scores on rank order for the NRMP match. J Gen Intern Med. 2021;36(11):3582–3. 33415597 10.1007/s11606-020-06370-4 PMC8606353

[61] Pilon S, Tandberg D. Neural network and linear regression models in residency selection. Am J Emerg Med. 1997;15(4):361–4. 9217525 10.1016/s0735-6757(97)90125-x

[62] Ortiz AV, Feldman MJ, Yengo-Kahn AM, Roth SG, Dambrino RJ, Chitale RV, et al. Words matter: using natural Language processing to predict neurosurgical residency match outcomes. J Neurosurg. 2023;138(2):559–66. 35901704 10.3171/2022.5.JNS22558

[63] Mahtani AU, Reinstein I, Marin M, Burk-Rafel J. A new tool for holistic residency application review: using natural Language processing of applicant experiences to predict interview invitation. Acad Med. 2023;98(9):1018–21. 36940395 10.1097/ACM.0000000000005210

[64] Johnstone RE, Neely G, Sizemore DC. Artificial intelligence software can generate residency application personal statements that program directors find acceptable and difficult to distinguish from applicant compositions. J Clin Anesth. 2023;89:111185. 37336139 10.1016/j.jclinane.2023.111185

[65] Patel V, Deleonibus A, Wells MW, Bernard SL, Schwarz GS. Distinguishing authentic voices in the age of ChatGPT: comparing AI-Generated and Applicant-Written personal statements for plastic surgery residency application. Ann Plast Surg. 2023;91(3):324–5. 37566815 10.1097/SAP.0000000000003653

[66] Yi PK, Ray ND, Segall N. A novel use of an artificially intelligent chatbot and a live, synchronous virtual question-and answer session for fellowship recruitment. BMC Med Educ. 2023;23(1):152. 36906574 10.1186/s12909-022-03872-z PMC10006550

[67] Zhao XX, Wu SP, Wang JY, Gong XY, He XR, Xi MJ et al. Comparison of Multiple Quantitative Evaluation Indices of Theoretical Knowledge and Clinical Practice Skills and Training of Medical Interns in Cardiovascular Imaging Using Blended Teaching and the Case Resource Network Platform (CRNP). Med Sci Monit Int Med J Exp Clin Res. 2020;26(dxw, 9609063):e923836. 10.12659/MSM.923836 PMC7191953 32297597

[68] Merritt C, Glisson M, Dewan M, Klein M, Zackoff M. Implementation and evaluation of an artificial intelligence driven simulation to improve resident communication with primary care providers. Acad Pediatr. 2022;22(3):503–5. 34923145 10.1016/j.acap.2021.12.013

[69] Webb JJ. Proof of Concept: Using ChatGPT to Teach Emergency Physicians How to Break Bad News. Cureus. 2023 May 9 [cited 2024 May 18]; Available from: https://www.cureus.com/articles/154391-proof-of-concept-using-chatgpt-to-teach-emergency-physicians-how-to-break-bad-news 10.7759/cureus.38755 PMC10250131 37303324

[70] El Saadawi GM, Tseytlin E, Legowski E, Jukic D, Castine M, Fine J, et al. A natural Language intelligent tutoring system for training pathologists: implementation and evaluation. Adv Health Sci Educ. 2008;13(5):709–22. 10.1007/s10459-007-9081-3 PMC2753375 17934789

[71] Kelahan LC, Fong A, Ratwani RM, Filice RW. Call case dashboard: tracking R1 exposure to High-Acuity cases using natural Language processing. J Am Coll Radiol. 2016;13(8):988–91. 27162046 10.1016/j.jacr.2016.03.012

[72] Lin H, Yang X, Wang WA, Content-Boosted. Collaborative filtering algorithm for personalized training in interpretation of radiological imaging. J Digit Imaging. 2014;27(4):449–56. 24526520 10.1007/s10278-014-9678-z PMC4090405

[73] Muntean GA, Groza A, Marginean A, Slavescu RR, Steiu MG, Muntean V, et al. Artificial intelligence for personalised ophthalmology residency training. J Clin Med. 2023;12(5):1825. 36902612 10.3390/jcm12051825 PMC10002549

[74] Chen H, Gangaram V, Shih G. Developing a more responsive radiology resident dashboard. J Digit Imaging. 2019;32(1):81–90. 30264216 10.1007/s10278-018-0123-6 PMC6382641

[75] Nori H, King N, McKinney SM, Carignan D, Horvitz E. Capabilities of GPT-4 on Medical Challenge Problems. 2023.

[76] Ali R, Tang OY, Connolly ID, Fridley JS, Shin JH, Zadnik Sullivan PL, et al. Performance of ChatGPT, GPT-4, and Google bard on a neurosurgery oral boards Preparation question bank. Neurosurgery. 2023;93(5):1090–8. 37306460 10.1227/neu.0000000000002551

[77] Bartoli A, May AT, Al-Awadhi A, Schaller K. Probing artificial intelligence in neurosurgical training: ChatGPT takes a neurosurgical residents written exam. Brain Spine. 2024;4:102715. 38163001 10.1016/j.bas.2023.102715 PMC10753430

[78] Gupta R, Park JB, Herzog I, Yosufi N, Mangan A, Firouzbakht PK, et al. Applying GPT-4 to the plastic surgery inservice training examination. J Plast Reconstr Aesthet Surg. 2023;87:78–82. 37812847 10.1016/j.bjps.2023.09.027

[79] Humar P, Asaad M, Bengur FB, Nguyen V. ChatGPT is equivalent to First-Year plastic surgery residents: evaluation of ChatGPT on the plastic surgery In-Service examination. Aesthet Surg J. 2023;43(12):NP1085–9. 37140001 10.1093/asj/sjad130

[80] Holmes J, Ye S, Li Y, Wu SN, Liu Z, Zhao H et al. Evaluating Large Language Models in Ophthalmology.

[81] Cohen A, Alter R, Lessans N, Meyer R, Brezinov Y, Levin G. Performance of ChatGPT in Israeli Hebrew OBGYN National residency examinations. Arch Gynecol Obstet. 2023;308(6):1797–802. 37668790 10.1007/s00404-023-07185-4

[82] Rizzo MG, Cai N, Constantinescu D. The performance of ChatGPT on orthopaedic in-service training exams: A comparative study of the GPT-3.5 turbo and GPT-4 models in orthopaedic education. J Orthop. 2024;50:70–5. 38173829 10.1016/j.jor.2023.11.056 PMC10758621

[83] Lum ZC. Can artificial intelligence pass the American board of orthopaedic surgery examination?? orthopaedic residents versus ChatGPT. Clin Orthop. 2023;481(8):1623–30. 37220190 10.1097/CORR.0000000000002704 PMC10344569

[84] Smith J, Choi PM, Buntine P. Will code one day run a code? Performance of Language models on ACEM primary examinations and implications. Emerg Med Australas. 2023;35(5):876–8. 37414729 10.1111/1742-6723.14280

[85] Mahajan AP, Shabet CL, Smith J, Rudy SF, Kupfer RA, Bohm LA. Assessment of Artificial Intelligence Performance on the Otolaryngology Residency In-Service Exam. OTP Open. 2023;7(4). Available from: https://www.scopus.com/inward/record.uri?eid=2-s2.0-85178235724&doi=10.1002%2foto2.98&partnerID=40&md5=d472c848d17df9629bd2685f1dc57c32 10.1002/oto2.98 PMC10687376 38034065

[86] Cheung BHH, Lau GKK, Wong GTC, Lee EYP, Kulkarni D, Seow CS et al. J Wang editor 2023 ChatGPT versus human in generating medical graduate exam multiple choice questions—A multinational prospective study (Hong Kong S.A.R., Singapore, Ireland, and the united Kingdom). PLoS ONE 18 8 e0290691. 10.1371/journal.pone.0290691 PMC10464959 37643186

[87] Wu JT, Wong KCL, Gur Y, Ansari N, Karargyris A, Sharma A, et al. Comparison of chest radiograph interpretations by artificial intelligence algorithm vs radiology residents. JAMA Netw Open. 2020;3(10):e2022779. 33034642 10.1001/jamanetworkopen.2020.22779 PMC7547369

[88] Yi PH, Kim TK, Yu AC, Bennett B, Eng J, Lin CT. Can AI outperform a junior resident? Comparison of deep neural network to first-year radiology residents for identification of pneumothorax. Emerg Radiol. 2020;27(4):367–75. 32643070 10.1007/s10140-020-01767-4

[89] Zhao C, Xiao M, Liu H, Wang M, Wang H, Zhang J, et al. Reducing the number of unnecessary biopsies of US-BI-RADS 4a lesions through a deep learning method for residents-in-training: a cross-sectional study. BMJ Open. 2020;10(6):e035757. 10.1136/bmjopen-2019-035757 PMC7282415 32513885

[90] Homayounieh F, Digumarthy S, Ebrahimian S, Rueckel J, Hoppe BF, Sabel BO, et al. An artificial Intelligence–Based chest X-ray model on human nodule detection accuracy from a multicenter study. JAMA Netw Open. 2021;4(12):e2141096. 34964851 10.1001/jamanetworkopen.2021.41096 PMC8717119

[91] Han SS, Park I, Eun Chang S, Lim W, Kim MS, Park GH, et al. Augmented intelligence dermatology: deep neural networks empower medical professionals in diagnosing skin Cancer and predicting treatment options for 134 skin disorders. J Invest Dermatol. 2020;140(9):1753–61. 32243882 10.1016/j.jid.2020.01.019

[92] Feng Y, Sim Zheng Ting J, Xu X, Bee Kun C, Ong Tien En E et al. Irawan Tan Wee Jun H,. Deep Neural Network Augments Performance of Junior Residents in Diagnosing COVID-19 Pneumonia on Chest Radiographs. Diagnostics. 2023;13(8):1397. 10.3390/diagnostics13081397 PMC10137770 37189498

[93] Olsson S, Ohlsson M, Öhlin H, Dzaferagic S, Nilsson M, Sandkull P, et al. Decision support for the initial triage of patients with acute coronary syndromes. Clin Physiol Funct Imaging. 2006;26(3):151–6. 16640509 10.1111/j.1475-097X.2006.00669.x

[94] Marchetti MA, Liopyris K, Dusza SW, Codella NCF, Gutman DA, Helba B, et al. Computer algorithms show potential for improving dermatologists’ accuracy to diagnose cutaneous melanoma: results of the international skin imaging collaboration 2017. J Am Acad Dermatol. 2020;82(3):622–7. 31306724 10.1016/j.jaad.2019.07.016 PMC7006718

[95] Paul SK, Kim CU, Shieh D, Zhou XY, Pan I, Mehra AA et al. Impact of an Artificial Intelligence Algorithm on Diabetic Retinopathy Grading by Ophthalmology Residents. medRxiv. 2023;((Paul S.K., samantha.paul2@uhhospitals.org; Kim C.U.; Shieh D.; Mehra A.A.; Sobol W.M.) Department of Ophthalmology, University Hospitals Cleveland Medical Center, Case Western Reserve University, School of Medicine, Cleveland, OH, United States(Zhou X.Y.). Available from: https://www.embase.com/search/results?subaction=viewrecord&id=L2026945804&from=export

[96] Fang Z, Xu Z, He X, Han W. Artificial intelligence-based pathologic myopia identification system in the ophthalmology residency training program. Front Cell Dev Biol. 2022;10:1053079. 36407106 10.3389/fcell.2022.1053079 PMC9669055

[97] Lee JH, Ha EJ, Kim D, Jung YJ, Heo S, Jang Y, ho, et al. Application of deep learning to the diagnosis of cervical lymph node metastasis from thyroid cancer with CT: external validation and clinical utility for resident training. Eur Radiol. 2020;30(6):3066–72. 32065285 10.1007/s00330-019-06652-4

[98] Chassagnon G, Billet N, Rutten C, Toussaint T, Cassius De Linval Q, Collin M, et al. Learning from the machine: AI assistance is not an effective learning tool for resident education in chest x-ray interpretation. Eur Radiol. 2023;33(11):8241–50. 37572190 10.1007/s00330-023-10043-1

[99] Shah C, Davtyan K, Nasrallah I, Bryan RN, Mohan S. Artificial Intelligence-Powered clinical decision support and simulation platform for radiology trainee education. J Digit Imaging. 2022;36(1):11–6. 36279026 10.1007/s10278-022-00713-9 PMC9590389

[100] Thanawala R, Jesneck J, Shelton J, Rhee R, Seymour NE. Overcoming systems factors in case logging with artificial intelligence tools. J Surg Educ. 2022;79(4):1024–30. 35193831 10.1016/j.jsurg.2022.01.013

[101] Gong JJ, Soleimani H, Murray SG, Adler-Milstein J. Characterizing styles of clinical note production and relationship to clinical work hours among first-year residents. J Am Med Inf Assoc. 2021;29(1):120–7. 10.1093/jamia/ocab253 PMC8714268 34963142

[102] Gao Y, Gu L, Wang Y, Wang Y, Yang F. Constructing a Chinese electronic medical record corpus for named entity recognition on resident admit notes. BMC Med Inf Decis Mak. 2019;19(S2):56. 10.1186/s12911-019-0759-2 PMC6454673 30961596

[103] Ouyang Y, Wu Y, Wang H, Zhang C, Cheng F, Jiang C, et al. Leveraging historical medical records as a proxy via multimodal modeling and visualization to enrich medical diagnostic learning. IEEE Trans Vis Comput Graph. 2024;30(1):1238–48. 37874707 10.1109/TVCG.2023.3326929

[104] Dimitroyannis R, Thodupunoori S, Polster SP, Das P, Roxbury CR. Residency education practices in endoscopic skull base surgery. J Neurol Surg Part B Skull Base. 2023;a–2226. 10.1055/a-2226-8294 PMC11495901 39444777

[105] Andrews J, Chartash D, Hay S. Gender bias in resident evaluations: natural Language processing and competency evaluation. Med Educ. 2021;55(12):1383–7. 34224606 10.1111/medu.14593

[106] Brunyé TT, Booth K, Hendel D, Kerr KF, Shucard H, Weaver DL, et al. Machine learning classification of diagnostic accuracy in pathologists interpreting breast biopsies. J Am Med Inf Assoc. 2024;31(3):552–62. 10.1093/jamia/ocad232 PMC10873842 38031453

[107] DiPietro R, Ahmidi N, Malpani A, Waldram M, Lee GI, Lee MR, et al. Segmenting and classifying activities in robot-assisted surgery with recurrent neural networks. Int J Comput Assist Radiol Surg. 2019;14(11):2005–20. 31037493 10.1007/s11548-019-01953-x

[108] Mathematics and Science Academy at the University of Texas Rio Grande Valley, Edinburg TX, Tippur A. Bridging the Gap: Integrating Artificial Intelligence into Medical Education. DHR Proc. 2023 [cited 2025 Jan 22]; Available from: https://dhrproceedings.org/index.php/DHRP/article/view/94/58

[109] Southworth J, Migliaccio K, Glover J, Glover J, Reed D, McCarty C, et al. Developing a model for AI across the curriculum: transforming the higher education landscape via innovation in AI literacy. Comput Educ Artif Intell. 2023;4:100127.

[110] Abbott KL, George BC, Sandhu G, Harbaugh CM, Gauger PG, Ötleş E, et al. Natural Language processing to estimate clinical competency committee ratings. J Surg Educ. 2021;78(6):2046–51. 34266789 10.1016/j.jsurg.2021.06.013

[111] Ötleş E, Kendrick DE, Solano QP, Schuller M, Ahle SL, Eskender MH, et al. Using natural Language processing to automatically assess feedback quality: findings from 3 surgical residencies. Acad Med. 2021;96(10):1457–60. 33951682 10.1097/ACM.0000000000004153

[112] Booth GJ, Ross B, Cronin WA, McElrath A, Cyr KL, Hodgson JA, et al. Competency-Based assessments: leveraging artificial intelligence to predict subcompetency content. Acad Med. 2023;98(4):497–504. 36477379 10.1097/ACM.0000000000005115

[113] Brown DC, Gonzalez-Vargas JM, Tzamaras HM, Sinz EH, Ng PK, Yang MX, et al. Evaluating the impact of assessment metrics for simulated central venous catheterization training. Simul Healthc J Soc Simul Healthc. 2024;19(1):27–34. 10.1097/SIH.0000000000000704 PMC10185707 36378597

[114] Ebina K, Abe T, Hotta K, Higuchi M, Furumido J, Iwahara N, et al. Objective evaluation of laparoscopic surgical skills in wet lab training based on motion analysis and machine learning. Langenbecks Arch Surg. 2022;407(5):2123–32. 35394212 10.1007/s00423-022-02505-9 PMC9399206

[115] Gates RS, Marcotte K, Moreci R, George BC, Kim GJ, Kraft KH, et al. Association of gender and operative feedback quality in surgical residents. J Surg Educ. 2023;80(11):1516–21. 37385931 10.1016/j.jsurg.2023.06.004

[116] Hernández-Rodríguez J, Rodríguez-Conde MJ, Santos-Sánchez JÁ, Cabrero-Fraile FJ. Development and validation of an educational software based in artificial neural networks for training in radiology (JORCAD) through an interactive learning activity. Heliyon. 2023;9(4):e14780. 37025816 10.1016/j.heliyon.2023.e14780 PMC10070709

[117] Jalali S, Stroulia E, Foster S, Persad A, Shi D, Forgie S, LiveBook. Competence Assessment with Virtual-Patient Simulations. In: 2017 IEEE 30th International Symposium on Computer-Based Medical Systems (CBMS). Thessaloniki: IEEE; 2017 [cited 2024 May 18]. pp. 47–52. Available from: http://ieeexplore.ieee.org/document/8104155/

[118] Madhavan R, Tang C, Bhattacharya P, Delly F, Basha MM. Evaluation of Documentation patterns of trainees and supervising physicians using data mining. J Grad Med Educ. 2014;6(3):577–80. 26279789 10.4300/JGME-D-13-00267.1 PMC4535228

[119] Vasoya MM, Shivakumar A, Pappu S, Murphy CP, Pei Y, Bricker DA, et al. ReadMI: an innovative app to support training in motivational interviewing. J Grad Med Educ. 2019;11(3):344–6. 31210875 10.4300/JGME-D-18-00839.1 PMC6570458

